# Histidine triad nucleotide‐binding protein 2 attenuates doxorubicin‐induced cardiotoxicity through restoring lysosomal function and promoting autophagy in mice

**DOI:** 10.1002/mco2.70075

**Published:** 2025-02-17

**Authors:** Hao Jiang, Jinyan Zhang, Daile Jia, Liwei Liu, Jinfeng Gao, Beijian Zhang, Zhen Dong, Xiaolei Sun, Wenlong Yang, Tiantong Ou, Suling Ding, Luna He, Yiqin Shi, Kai Hu, Aijun Sun, Junbo Ge

**Affiliations:** ^1^ Department of Cardiology Zhongshan Hospital Fudan University Shanghai Institute of Cardiovascular Diseases Shanghai Shanghai China; ^2^ National Clinical Research Center for Interventional Medicine Shanghai China; ^3^ State Key Laboratory of Cardiovascular Diseases Zhongshan Hospital Fudan University Shanghai China; ^4^ NHC Key Laboratory of Ischemic Heart Diseases Shanghai China; ^5^ Key Laboratory of Viral Heart Diseases Chinese Academy of Medical Sciences Shanghai China; ^6^ Department of Nephrology Zhongshan Hospital Fudan University Shanghai China; ^7^ Institutes of Biomedical Sciences Fudan University Shanghai China

**Keywords:** autophagy, doxorubicin, HINT2, lysosome, NAD^+^/NADH

## Abstract

Doxorubicin (DOX) is an effective chemotherapy drug widely used against various cancers but is limited by severe cardiotoxicity. Mitochondria–lysosome interactions are crucial for cellular homeostasis. This study investigates the role of histidine triad nucleotide‐binding protein 2 (HINT2) in DOX‐induced cardiotoxicity (DIC). We found that HINT2 expression was significantly upregulated in the hearts of DOX‐treated mice. Cardiac‐specific *Hint2* knockout mice exhibited significantly worse cardiac dysfunction, impaired autophagic flux, and lysosomal dysfunction after DOX treatment. Mechanistically, HINT2 deficiency reduced oxidative phosphorylation complex I activity and disrupted the nicotinamide adenine dinucleotide NAD^+^/NADH ratio, impairing lysosomal function. Further, HINT2 deficiency suppressed sterol regulatory element binding protein 2 activity, downregulating transcription factor A mitochondrial, a critical regulator of complex I. Nicotinamide mononucleotide (NMN) supplementation restored lysosomal function in vitro, while cardiac‐specific *Hint2* overexpression using adeno‐associated virus 9 or adenovirus alleviated DIC both in vivo and in vitro. These findings highlight HINT2 as a key cardioprotective factor that mitigates DIC by restoring the NAD^+^/NADH ratio, lysosomal function, and autophagy. Therapeutic strategies enhancing HINT2 expression or supplementing NMN may reduce cardiac damage and heart failure caused by DOX.

## Graphical abstract

Proposed model of how HINT2 deficiency aggravates DOX‐induced cardiotoxicity

*Hint2* cKO causes cholesterol accumulation and subsequently inhibits the transcriptional activity of SREBF2, which downregulates TFAM expression. The decrease in TFAM expression reduces both the expression and activity of OXPHOS complex Ι, and lowers the NAD^+^/NADH ratio, leading to lysosome dysfunction. This impaired lysosome function results in the accumulation of autolysosomes and dysfunction in autolysosomes acidification, which subsequently increases cardiotoxicity.

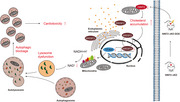

## 1 INTRODUCTION

Doxorubicin (DOX) is widely used in treating hematological malignancies and solid tumors. However, the clinical use of DOX is often limited by its severe acute and chronic cardiotoxicity.^1^ Understanding the mechanisms and developing protective strategies against DOX‐induced cardiotoxicity (DIC) have been a focus of recent research.

DIC is primarily driven by oxidative stress, iron accumulation, and mitochondrial dysfunction in cardiomyocytes. The formation of reactive oxygen species (ROS) via DOX–iron complexes results in lipid peroxidation and cellular damage.^2^, ^3^ Dexrazoxane, an iron‐chelating agent, has been proven effective in mitigating these effects by reducing mitochondrial iron accumulation and oxidative stress.^4^, ^5^ Additionally, DOX targets topoisomerase IIβ, causing DNA double‐strand breaks and apoptosis. Emerging research highlights ferroptosis, a regulated iron‐dependent cell death process characterized by lipid peroxidation, as a key pathway in DIC.^6^, ^7^ Endothelial dysfunction also contributes to DIC, through mechanisms such as impaired vascularization,^8^ increased microvascular permeability,^9^ and disrupted endothelial paracrine signaling.^10^ Furthermore, DOX has been shown to disrupt autophagy, increase mitochondrial apoptosis, and eventually impede mitochondrial metabolic circuits.^11^, ^12^ Recent studies have emphasized the essential role of mitochondria–lysosome interaction in maintaining cellular homeostasis and have shown that autophagic flux is impaired in DIC.^13^ However, the molecular elements connecting mitochondria and lysosomes in the context of DIC remain elusive.

Through mass spectrometry, we identified the mitochondrial protein histidine triad nucleotide‐binding protein 2 (HINT2), which exhibited a significant increase in DOX‐treated heart samples. HINT2, a member of the HINT superfamily, is primarily located in the mitochondria.^14^ Global *Hint2* knockout mice exhibit impaired glutamate dehydrogenase activity, hyperacetylated mitochondrial proteins, glucose metabolism disorder, and cholesterol accumulation in the liver, mirroring hepatic phenotypes observed in sirtuins 3 (*SIRT3*) knockout mice. Furthermore, cardiac‐specific overexpression of HINT2 induced by adeno‐associated virus 9 (AAV9), has been shown to improve the nicotinamide adenine dinucleotide NAD^+^/NADH ratio in the hearts of postmyocardial infarction mice.^15^ These findings suggest that HINT2 may play a role in maintaining NAD^+^ homeostasis in the heart. However, the specific role of HINT2 in DIC, particularly its impact on NAD^+^ homeostasis, remains to be fully elucidated.

In this study, we investigated the role of HINT2 in the development of cardiotoxicity induced by a single high dose (15 mg/kg) and multiple low doses (5 mg/kg/week for 4 weeks) of DOX. Using a cardiac‐specific *Hint2* knockout mice (cKO) mouse model, we examined autophagic flux following DOX treatment in the absence of HINT2, focusing particularly on the NAD^+^/NADH ratio and lysosomal function.

## 2 RESULTS

### 2.1 HINT2 is upregulated in DOX‐treated mice and cKO exacerbates DIC

**FIGURE 1:**
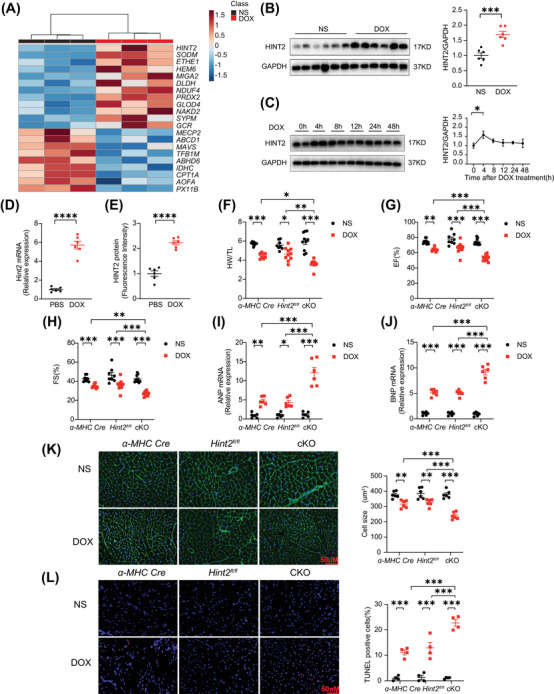
HINT2 is upregulated in hearts of mice after DOX treatment and HINT2 deficiency exacerbates the heart function of mice after DOX treatment. (A) Heatmap of the 21 proteins that significantly changed in heart samples, as analyzed by mass spectrometry (7 days after 15 mg/kg DOX, i.p., single injection, *n *= 3 mice/group). (B) Representative gel blots and quantification showing levels of HINT2 in heart samples (7 days after 15 mg/kg DOX, i.p., single injection, *n *= 6 mice/group). (C) Time course of representative gel blots and quantification showing levels of HINT2 in AMCMs (*n *= 4 wells/group). (D) Quantification of mRNA expression of *Hint2* of hiPSC‐CMs after DOX treatment (1 µM, 4 h) (*n *= 6 wells/group). (E) quantification showing levels of HINT2 fluorescence intensity of hiPSC‐CMs after DOX treatment (*n *= 6 samples/group). (F, G, and H) Quantification of HW/TL (*n *= 8–11 mice/group), EF and FS (*n *= 8–12 mice/group) of mice at 7th day. (I and J) Quantification of mRNA expression of *Anp* and *Bnp* of mice heart samples at 7th day (*n *= 6 mice/group). (K) Representative images and quantification of heart sections from mice were stained with WGA to demarcate the cell boundaries (*n *= 6 mice/group). (L) Representative images and quantification of TUNEL staining of heart samples from mice (*n *= 4 mice/group). Data are mean ± SEM. *p < 0.05, **p < 0.01, ***p < 0.001, ****p < 0.0001. *p* Values are calculated by unpaired Student's *t*‐test or two‐way ANOVA followed by Tukey's multiple comparisons test.

To investigate the mechanisms of DIC, we performed unbiased mass spectrometry on heart tissues from mice treated with DOX (15 mg/kg, i.p., single injection) (Figure S1A). This revealed 975 differentially expressed proteins (*p* < 0.05, fold change > 1.5 or <1/1.5) in the heart tissues of DOX‐treated mice compared with those treated with normal saline (NS). Gene Ontology (GO) enrichment analysis indicated a close relationship between mitochondria and myocardial damage induced by DOX (Figure S1B). To exclude noncardiomyocyte influence, we used mRNA expression profiles (GSE42177) from post‐DOX‐treated neonatal rat cardiac myocytes samples. This revealed 152 genes with consistent trends in both the mass spectrometry and GSE42177 mRNA expression profiles. Given the role of mitochondria in DIC, we selected 33 mitochondrial‐related genes from the total of 152 genes and cross‐referenced them with DOX‐related genes in the Comparative Toxicogenomics Database (http://ctdbase.org/). This yielded 21 differential genes, with *Hint2* being the most significantly altered gene (Figures 1A and S1C). Western blot showed that HINT2 expression was significantly increased in DOX‐treated mice (7 days after 15 mg/kg DOX, i.p.) (Figure 1B) and in isolated cardiomyocytes from 8 to 10‐week‐old C57BL/6J mice 4 h after DOX treatment (Figure 1C). In primary cardiomyocytes induced from human‐specific induced pluripotent stem cells (hiPSC‐CMs), DOX treatment (1 µM for 4 h) significantly upregulated *Hint2* mRNA expression (Figure 1D), and immunofluorescence analysis further confirmed a marked increase in HINT2 protein levels (Figures 1E and S1D).

To study the role of HINT2 in cardiomyocytes, we generated cKO mice by crossing *Hint2*‐floxed (*Hint2^fl/fl^*) with an *alpha myosin heavy chain* (*α‐MHC*) *Cre* recombinase‐expressing transgenic line (Figure S2A,B). Subsequent results showed that heart weight/tibia length ratios (HW/TL), ejection fraction (EF), and fractional shortening (FS) were significantly lower in cKO DOX‐treated mice than in control mice (*α‐MHC‐Cre* and *Hint2^fl/fl^* DOX‐treated mice) (Figure 1F,G,H). Atrial natriuretic peptide (ANP) and B‐type natriuretic peptide (BNP) mRNA expression levels were significantly higher in cKO mice than in control mice (Figure 1I,J). Cardiac atrophy, as assessed by wheat germ agglutinin (WGA), was also more severe in DOX‐treated cKO mice (Figure 1K). These changes were accompanied by significantly increased TUNEL‐positive cells in heart tissue and *Hint2*‐knockdown significantly increased apoptosis in primary neonatal mouse ventricular myocytes (NMVM) compared with controls after DOX treatment (1 µM for 4 h) (Figures 1L and S3A). We found upregulated hyperacetylation of mitochondrial proteins in heart tissue of cKO mice compared with *Hint2^fl/fl^* with similar levels of protein expression of enzymes involved in glucose glycolysis, glucose oxidation, and lipid metabolism among groups (Figure S3B,C). The implications of these findings on DIC were not examined in detail and should be explored in future studies. To extend the clinical significance of our study, mice were injected with a low dose of DOX (5 mg/kg/week) for four successive doses over 28 days (Figure S4A). cKO also exacerbated cardiac dysfunction in the chronic low‐dose DIC mouse model, as indicated by lower EF and FS values (Figure S4B,C).

Furthermore, cardiomyocytes were isolated from 8‐ to 10‐week‐old *Hint2^fl/fl^* mice and cKO mice. Subsequently, these adult mouse cardiomyocytes (AMCMs) were treated with DOX (1 µM, 4 h) or PBS. The resting cell length of the cardiomyocytes was similar across all groups (Figure S4D). Cardiomyocytes isolated from cKO mice exhibited more severe contraction impairment after DOX treatment compared with those from *Hint2^fl/fl^* mice, as evidenced by significant reductions in both the range and velocity of contraction (Figure S4E,F,G). Diastolic function, as indicated by +dL/dt, was also significantly compromised in cKO cardiomyocytes, and TR50 was marginally prolonged following DOX treatment (Figure S4H,I). Following DOX treatment, the number of Calcein‐AM‐positive live cells significantly decreased after *Hint2* knockout, and the number of propidium iodide‐positive dead cells increased in AMCMs isolated from cKO mice (Figure S4J). *Hint2* knockout resulted in an upregulation of cleaved caspase‐3 in response to DOX treatment in vitro (Figure S4K).

### 2.2 *Hint2* cardiac‐specific knockout contributed to the accumulation of autolysosomes in the heart following DOX treatment (15 mg/kg, single, i.p.)

**FIGURE 2:**
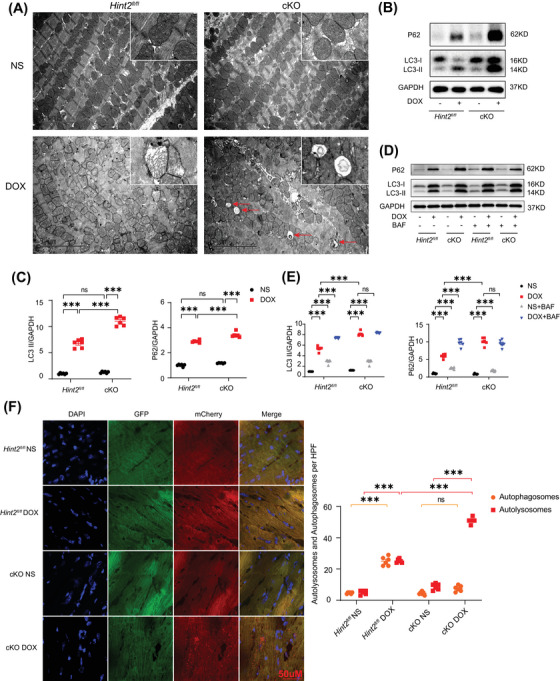
HINT2 deficiency impairs autophagic flux after DOX treatment. (A) Representative images of heart samples by TEM in (×1400) magnification (red arrows indicate autophagosome or autolysosome). (B and C) Representative gel blots and quantification showing levels of LC3‐II and P62 in heart samples from mice (7 days after 15 mg/kg DOX, i.p., single injection, *n *= 6 mice/group). (D and E) Representative gel blots and quantification showing levels of LC3‐II and P62 in heart samples from mice (7 days after 15 mg/kg DOX, i.p., single injection; BAF 1.5 mg/kg was injected intraperitoneally 2 h before death, *n *= 6 mice/group). (F) Representative images of AAV9–mCherry–GFP–LC3 puncta in the heart tissue of mice and quantification of average number of autophagosomes (yellow dots) and autolysosomes (red dots) (*n *= 6 mice/group, each value is the mean of 6 high‐power fields from each mouse). Data are mean ± SEM. *p < 0.05, **p < 0.01, ***p < 0.001. *p* Values are calculated by two‐way ANOVA followed by Tukey's multiple comparisons test.

Given that HINT2 is primarily located in the mitochondria,^14^ we assessed the effects of HINT2 deficiency on mitochondrial morphology and dynamics. Mitochondria were labeled with MitoTracker™ Dyes for visualization. Compared with control AMCMs, DOX‐treated AMCMs showed a reduced mean mitochondrial size. However, cKO did not further affect mitochondrial size under DOX treatment (Figure S5A). Among the mitochondrial fission proteins (Drp1) and fusion proteins (Mfn1, Mfn2, and Opa1), only Mfn2 expression was significantly reduced in the DOX‐treated hearts (Figure S5B). Transmission electron microscopy revealed low electron density in the mitochondrial matrix, disordered cristae, and a significant accumulation of autophagosomes or autolysosomes in the heart tissue of cKO mice after DOX treatment (Figure 2A). Western blot analysis showed a significant increase in the expression of autophagy markers LC3‐II and P62 in DOX‐treated *Hint2^fl/fl^* mice, which was further increased in DOX‐treated cKO mice (Figure 2B,C). However, similar levels of P62 mRNA expression were observed in both *Hint2^fl/fl^* and cKO groups after DOX treatment (Figure S5C). Moreover, the ATG5 protein level, a key factor in autophagy initiation, remained unchanged between *Hint2^fl/fl^* and cKO mice after DOX treatment (Figure S5D). These data indicate that HINT2 does not affect autophagy initiation.

We then administered Bafilomycin A1 (BAF), an inhibitor of V‐ATPase‐dependent acidification, to each group in vivo.^16^ BAF (1.5 mg/kg) was injected intraperitoneally 2 h before sacrifice. BAF alone significantly increased LC3II and P62 protein expression in both *Hint2^fl/fl^* and cKO mice. LC3‐II and P62 were further significantly increased after BAF treatment in DOX‐treated *Hint2^fl/fl^* mice. However, LC3‐II and P62 levels remained unchanged upon BAF treatment in DOX‐treated cKO mice (Figure 2D,E), which indicates that cKO already impaired lysosomal function and thereby abrogating autophagic flux by affecting the degradation process.

To further investigate the autophagic flux process, mice were injected with AAV9–mCherry–GFP–LC3. Both autophagosomes (yellow dots) and autolysosomes (red dots) significantly increased in *Hint2^fl/fl^* mice after DOX treatment. Additionally, autolysosomes significantly increased in DOX‐treated cKO mice compared with DOX‐treated *Hint2^fl/fl^* mice, without a corresponding increase in autophagosomes (Figure 2F). The results from Figures 2D,F and S5D suggest that under DOX treatment, cardiac‐specific knockout of *Hint2* impairs lysosomal function, causes autolysosomes accumulation and thereby blocks the autolysosome degradation process.

### 2.3 HINT2 regulated lysosome function in AMCMs

**FIGURE 3:**
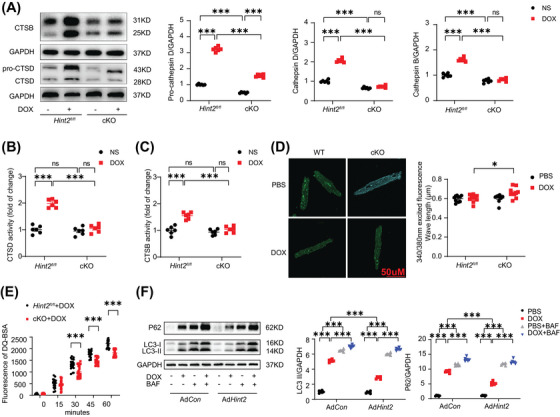
HINT2 deficiency impairs lysosome function after DOX treatment and HINT2 overexpression restores autophagy flux. (A) Representative gel blots and quantification showing levels of pro‐CTSD, CTSD, and CTSB in heart samples from mice (7 days after 15 mg/kg DOX, i.p., single injection, *n *= 6 mice/group). (B and C) Quantification of activity of CTSD and CTSB in heart samples from mice (7 days after 15 mg/kg DOX, i.p., single injection, *n *= 6 mice/group). (D) Representative images of AMCMs after DOX treatment (1 µM, 4 h) stained with LysoSensor Yellow/Blue DND‐160 and quantification. AMCMs were stained with LysoSensor Yellow/Blue DND‐160 (10 µM) for 20 min and detected with a plate reader at the excited fluorescence signal 340 and 380 nm (*n *= 10 wells from three independent experiments). (E) Quantification of DQ‐BSA hydrolysis rate of AMCMs loaded with bovine serum albumin labeled with a green BODIPY dye (*n *= 20 wells/group). (F) Representative gel blots and quantification showing levels of LC3‐II and P62 in AMCMs infected with Ad*Con* or Ad*Hint2* after PBS or DOX treatment (1 µM, 4 h), with or without BAF treatment (100 nM, 4 h before harvest, *n *= 6 wells/group). Data are mean ± SEM. *p < 0.05, **p < 0.01, ***p < 0.001. *p* Values are calculated by two‐way ANOVA followed by Tukey's multiple comparisons test.

Given that lysosomes are key cytoplasmic organelles responsible for autolysosome degradation, we hypothesize that HINT2 plays a crucial role in regulating lysosomal function. Cathepsin B (CTSB) and Cathepsin D (CTSD), two ubiquitously expressed lysosomal proteases involved in normal protein degradation,^17^, ^18^ exhibited significantly lower expression and activity in the hearts of cKO mice following DOX treatment (Figure 3A,B,C). However, knockout of *Hint2* did not affect the expression of either LAMP1 or LAMP2, indicating that the number of lysosomes was unchanged (Figure S5E). We also used Lysosensor Yellow/Blue DND‐160 to detect changes in lysosomal pH in DOX‐treated AMCMs (1 µM for 4 h). A higher 340/380 fluorescence ratio indicated that cardiac‐specific knockout of *Hint2* impaired lysosomal acidification after DOX treatment (Figure 3D). Furthermore, the DQ‐BSA hydrolysis rate was significantly reduced in AMCMs from DOX‐treated cKO mice, indicating impaired lysosomal proteolytic capacity (Figure 3E). Overexpression of HINT2 significantly reduced LC3‐II and P62 accumulation caused by DOX (1 µM for 4 h). Additionally, BAF treatment in Ad*Hint2* AMCMs led to further accumulation, indicating that HINT2 overexpression may enhance autolysosomal degradation (Figure 3F).

### 2.4 **HINT2 deficiency decreased NAD^+^/NADH ratio by impairing** oxidative phosphorylation complex I

**FIGURE 4:**
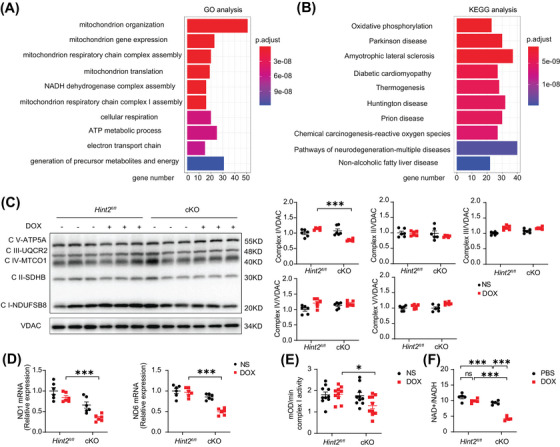
HINT2 deficiency impairs OXPHOS complex Ι and downregulates ratio of NAD^+^/NADH. (A and B) GO analysis and KEGG analysis of differentially expressed protein in heart samples from mice (7 days after 15 mg/kg DOX, i.p., single injection, 3 mice/group). (C) Representative gel blots and quantification showing levels of OXPHOS complexes Ι–V in heart samples from mice (7 days after 15 mg/kg DOX, i.p., single injection, *n *= 6 mice/group). (D) Quantification of mRNA levels of mtND1 and mtND6 of mice heart samples (7 days after 15 mg/kg DOX, i.p., single injection, *n *= 6 mice/group). (E) Quantification of activity of OXPHOS complex Ι in heart samples from mice (7 days after 15mg/kg DOX, i.p., single injection, *n *= 10 mice/group). (F) Quantification of ratio of NAD^+^/NADH in AMCMs after DOX treatment (1 µM, 4 h) (*n *= 5 wells/group). Data are mean ± SEM. *p < 0.05, **p < 0.01, ***p < 0.001. *p* Values are calculated by two‐way ANOVA followed by Tukey's multiple comparisons test.

To elucidate the underlying mechanism of impaired lysosomal function in cKO hearts after DOX treatment, mass spectrometry was conducted to identify differences in heart tissues from the left ventricles of *Hint2^fl/fl^* and cKO mice. Bioinformatics analysis revealed that HINT2 deficiency affects the mitochondrial respiratory chain and associated oxidative phosphorylation (OXPHOS) function (Figure 4A,B). Western blot analysis demonstrated that HINT2 deficiency reduced the expression of respiratory chain complex I but did not affect other complexes compared with *Hint2^fl/fl^* mice after DOX treatment (Figure 4C).

We further investigated complex I at the transcriptional level. HINT2 deficiency resulted in reduced mRNA levels of mitochondrial‐encoded subunits ND1 and ND6 after DOX treatment (Figure 4D), while the mRNA levels of nuclear‐encoded subunits in complex I remained unaffected (Figure S6A). Protein expression of subunits from complexes II, III, IV, and V remained unchanged after DOX treatment (Figure S6B). Moreover, mitochondria isolated from heart tissue of post‐DOX cKO mice exhibited diminished activity of respiratory chain complex I function indicating a reduced ability to oxidize NADH to NAD^+^ (Figure 4E). These findings suggest that cardiac‐specific knockout of *Hint2* impairs complex I expression and function. Given that a balanced NAD^+^/NADH ratio, a key parameter of complex I activity, is essential for maintaining lysosomal function and autophagic flux,^19^, ^20^ we examined the NAD^+^/NADH ratio in vitro. Results showed a significantly lower NAD^+^/NADH ratio in AMCMs isolated from cKO mice compared with those from *Hint2^fl/fl^* mice after DOX treatment (1 µM for 4 h) (Figure 4F). In summary, cardiac‐specific knockout of *Hint2* led to weakened complex I function and a lower NAD^+^/NADH ratio.

### 2.5 HINT2 deficiency impairs lysosomal function by influencing NAD^+^/NADH

**FIGURE 5:**
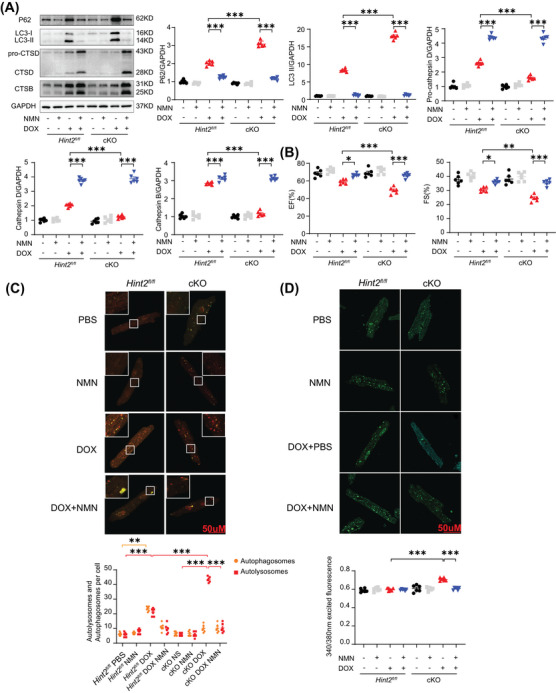
NMN could restore the autophagy flux in cKO mice treated with DOX. (A) Representative gel blots and quantification showing levels of LC3‐II, P62, pro‐CTSD, CTSD, and CTSB in heart samples from mice (*n *= 6 mice/group). (B) Quantification of EF and FS of mice (*n *= 6 mice/group). (C) Representative images of mCherry–GFP–LC3 puncta in the AMCMs and average number of autophagosomes (yellow dots) and autolysosomes (red dots) per cell after DOX treatment (1 µM, 4 h) with or without NMN replenishment (*n *= 6 samples/group, each value is the mean of six cells from each well). (D) Representative images and quantification of AMCMs stained with LysoSensor Yellow/Blue DND‐160 after DOX treatment (1 µM, 4 h) with or without NMN replenishment. AMCMs were stained with LysoSensor Yellow/Blue DND‐160 (10 µM) for 20 min and detected with a plate reader at the excited fluorescence signal 340 and 380 nm (*n *= 6 wells from three independent experiments). Data are mean ± SEM. *p < 0.05, **p < 0.01, ***p < 0.001. *p* Values are calculated by two‐way ANOVA followed by Tukey's multiple comparisons test.

Given that HINT2 primarily influences mitochondrial complex Ι and is associated with the NAD^+^/NADH ratio, we hypothesized that an imbalance in the NAD^+^/NADH ratio might cause lysosomal dysfunction in cKO mice after DOX treatment. We treated mice with nicotinamide mononucleotide (NMN), a precursor to NAD^+^, to determine whether modulating the NAD^+^/NADH ratio could restore autophagic flux and lysosomal function (Figure S7A). NMN treatment increased CTSD and CTSB expression and reduced P62 and LC3‐II accumulation in cKO mice after DOX treatment (Figure 5A). NMN treatment significantly preserved the EF and FS of *Hint2^fl/fl^* and cKO mice after DOX treatment, but not in *Hint2^fl/fl^* and cKO mice without DOX treatment (Figure 5B).

Consistent with improved cardiac function, NMN increased the live/death ratio of cKO AMCMs after DOX treatment (1 µM for 4 h) in vitro (Figure S7B). Consistent with P62 and LC3‐II expression, NMN reduced autolysosome accumulation and restored the autolysosome/autophagosome ratio after DOX treatment (1 µM for 4 h) in cKO AMCMs (Figure 5C). NMN replenishment also enhanced the lysosomal acidification capacity of AMCMs in vitro (Figure 5D). These results suggest that the mitochondrial protein HINT2 influences the NAD^+^/NADH ratio, thereby regulating lysosomal function.

### 2.6 **Hint2 knockout reduces OXPHOS complex I expression by inhibiting SREBF2 and subsequently downregulating** transcription factor A mitochondrial

**FIGURE 6:**
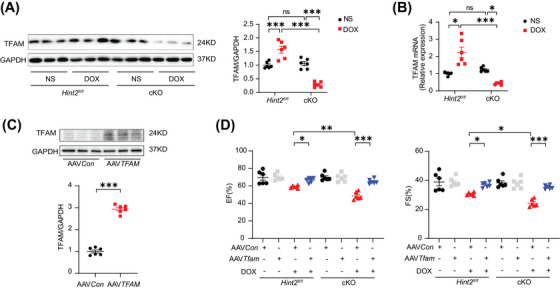
HINT2 deficiency downregulates TFAM expression and TFAM overexpression could restore cardiac dysfunction caused by HINT2 deficiency. (A) Representative gel blots and quantification showing levels of TFAM in heart samples from mice (*n *= 6 mice/group). (B) Quantification of mRNA levels of *TFAM* of mice heart samples (*n *= 6 mice/group). (C) Representative gel blots and quantification showing levels of TFAM in heart samples from mice (*n *= 6 mice/group). (D) Quantification of EF and FS of *Hint2^fl/fl^* and cKO mice injected with AAV*Con* or AAV*TFAM* at 7 days after NS or DOX treatment (*n *= 6 mice/group). Data are mean ± SEM. *p < 0.05, **p < 0.01, ***p < 0.001. *p* Values are calculated by two‐way ANOVA followed by Tukey's multiple comparisons test.

Considering the role of transcription factor A mitochondrial (TFAM) in regulating mtDNA transcription in complex I,^21^ we assessed TFAM at both protein and mRNA levels. Our findings revealed a significant reduction in both protein and mRNA expression in DOX‐treated cKO mice compared with DOX‐treated *Hint2^fl/fl^* mice (Figure 6A,B). To further investigate the impact of TFAM on cardiac function after DOX administration, we overexpressed TFAM using AAV9–*TFAM* in mice (Figure 6C). TFAM overexpression helped maintain cardiac function after DOX treatment in both DOX‐treated cKO and *Hint2^fl/fl^* mice (Figure 6D).

**FIGURE 7:**
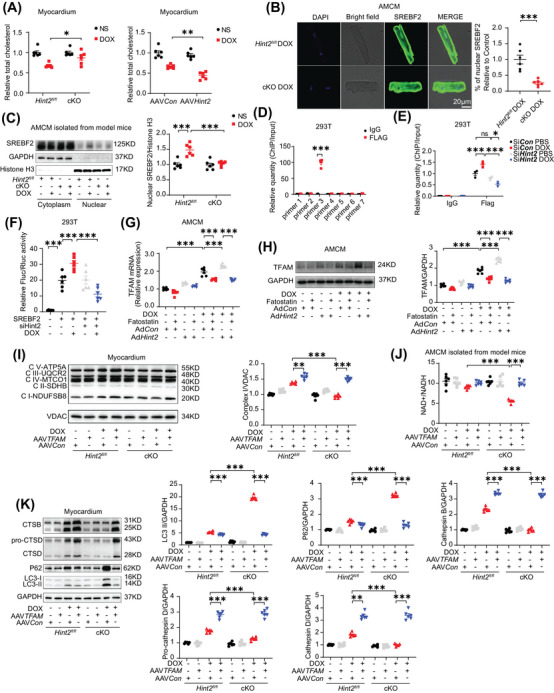
HINT2 deficiency cause cholesterol accumulation and decreases SREBF2 nuclear translocation which impairs *TFAM* transcription. A Quantification of cholesterol level of heart samples from mice (*n *= 6 mice/group). (B) Representative images and quantification of SREBF2 immunofluorescence analysis in AMCMs following DOX treatment (1 µM, 4 h) (*n *= 6 samples/group). (C) Representative gel blots and quantification showing levels of SREBF2 translocation in AMCMs from model mice (*n *= 6 mice/group). (D) ChIP analysis of SREBF2 at seven positions of *TFAM* promoter in HEK293T cells (*n *= 4 /group). (E) ChIP assays of SREBF2 binding to the *TFAM* promoter in HEK293T transfected with si*Con* or si*Hint2* and treated with or without DOX (1 µM, 4 h) (*n *= 3 wells/group). (F) Dual luciferase report assay used to detect *TFAM* promoter activity in HEK293T cells (*n *= 6 wells/group). (G) Quantification of mRNA levels of *TFAM* in AMCMs (DOX, 1 µM, 4 h; Fatostatin, 1 µM, 24 h) (*n *= 6 wells/group). (H) Representative gel blots and quantification showing levels of TFAM in AMCMs (DOX, 1 µM, 4 h; Fatostatin, 1 µM, 24 h) (*n *= 6 wells/group). (I and K) Representative gel blots and quantification showing levels of OXPHOS complexes Ι–V, LC3‐II, P62, pro‐CTSD, CTSD, and CTSB in heart samples from mice (*n *= 6 mice/group). (J) Quantification of ratio of NAD^+^/NADH in AMCMs from model mice after DOX treatment (1 µM, 4 h) (*n *= 6 mice/group). Data are mean ± SEM. *p < 0.05, **p < 0.01, ***p < 0.001. *p* Values are calculated by unpaired Student's *t* test or two‐way ANOVA followed by Tukey's multiple comparisons test.

Given that HINT2 has been identified as an important metabolic enzyme in the regulation of cholesterol metabolism,^22^ we hypothesized that HINT2 might modulate TFAM expression through a cholesterol‐dependent mechanism. Initially, we measured myocardial cholesterol levels and found a significant elevation in heart tissue from DOX‐treated cKO mice compared with DOX‐treated *Hint2^fl/fl^* mice. Subsequently, we overexpressed HINT2 in vivo using AAV9 carrying *Hint2* under the cTnT promoter. This resulted in a significant reduction in myocardial cholesterol levels in DOX‐treated AAV*Hint2* mice compared with DOX‐treated AAV*Con* mice (Figure 7A). Given that cholesterol can influence the transcriptional capability of SREBF1/2, a cholesterol sensor in the endoplasmic reticulum and nucleus, we investigated whether SREBF1/2 modulates TFAM expression.

The nuclear translocation of sterol regulatory element binding protein 2 (SREBF2), which indicates its transcriptional activity, was assessed by immunofluorescence in AMCMs. As expected, a decrease in nuclear translocation of SREBF2 was observed in cKO AMCMs after DOX treatment compared with controls (Figure 7B). Consistent with the immunofluorescence data, nuclear SREBF2 protein levels significantly increased after DOX treatment in AMCMs from model mice but were markedly reduced by *Hint2* knockout (Figure 7C). ChIP assay results showed that SREBF2 directly binds to the region between −1140 and −909 bp upstream of the TFAM transcriptional start site (Figure 7D). After siRNA was applied to knock down *Hint2* in HEK293T cells (Figure S8A), the transcriptional activity of *TFAM* by SREBF2 was reduced in *Hint2*‐knockdown cells (Figure 7E). We further confirmed HINT2's effect on SREBF2 transcriptional efficiency, particularly its binding to the *TFAM* promoter, using a dual luciferase reporter assay. The results showed that SREBF2 transcriptional activity significantly increased after DOX treatment in HEK293T cells but was substantially reduced by *Hint2* small interfering RNA (Figure 7F). In contrast, adenoviral overexpression of *Hint2* in vitro increased both mRNA and protein levels of TFAM, an effect counteracted by the SREBF2 inhibitor Fatostatin (Figure 7G,H).

Furthermore, using AAV9 carrying *TFAM*, cardiomyocyte‐specific *TFAM* overexpression reversed the downregulation of complex I level and NAD^+^/NADH ratio in heart tissue caused by HINT2 deficiency after DOX treatment (Figure 7I,J). Finally, cardiomyocyte‐specific *TFAM* overexpression rescued impaired lysosomal function, as indicated by the downregulation of CTSB, CTSD caused by HINT2 deficiency after DOX treatment (Figure 7K). These findings suggest a positive correlation between HINT2 and SREBF2 transcriptional activity. Furthermore, HINT2 seems to regulate mitochondrial complex I and subsequently NAD^+^/NADH ratio through the cholesterol–SREBF2–TFAM axis.

### 2.7 *Hint2* cardiac‐specific overexpression protects cardiac function after DOX treatment

**FIGURE 8:**
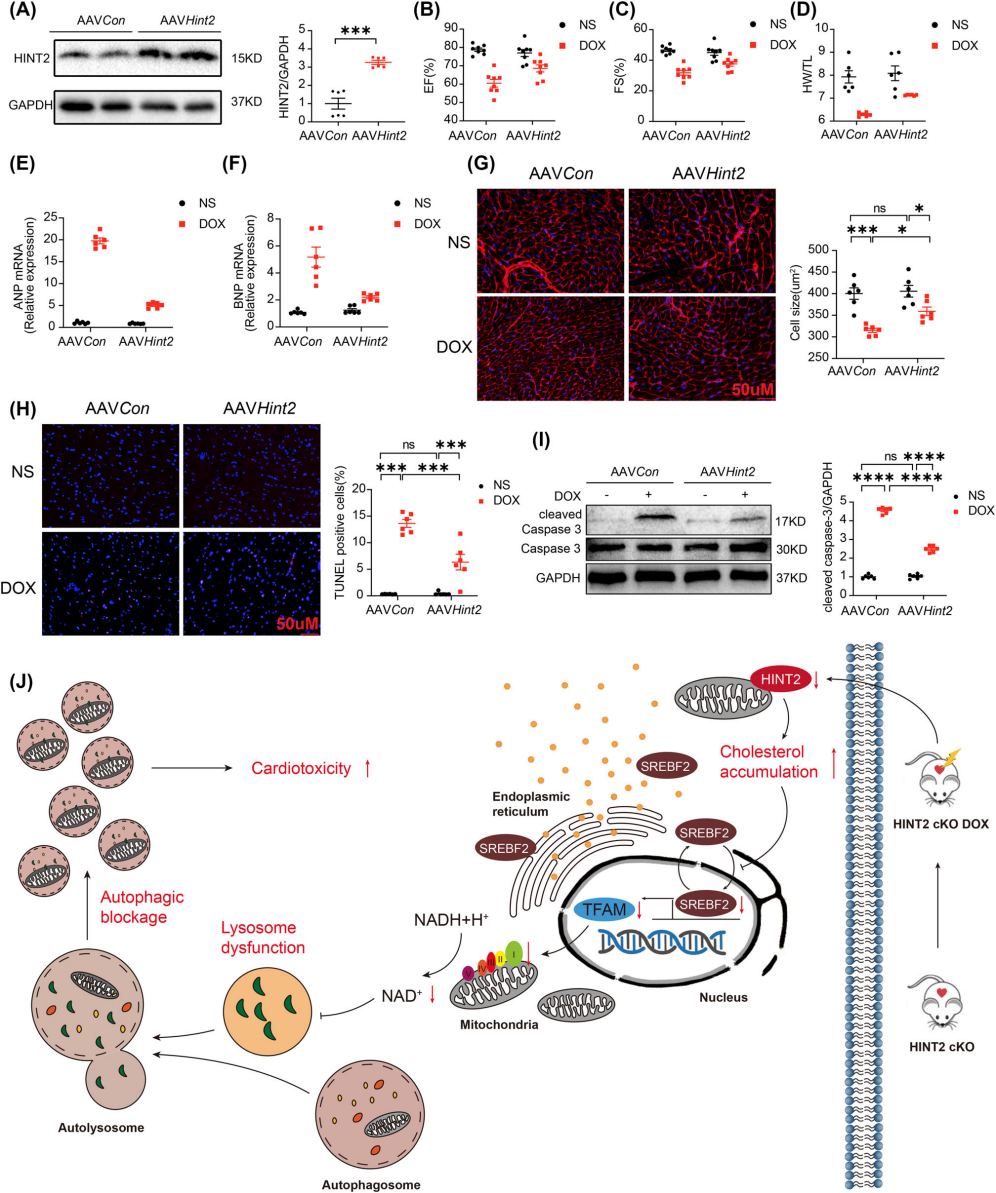
Cardiac specific overexpression of HINT2 attenuates DIC. (A) Representative gel blots and quantification showing levels of HINT2 in heart samples from mice (*n *= 6 mice/group). (B, C, and D) Quantification of EF, FS (*n *= 8 mice/group) and HW/TL (*n *= 6 mice/group) of mice. (E and F) Quantification of mRNA expression of *Anp* and *Bnp* of heart samples from mice (*n *= 6 mice/group). (G) Representative images and quantification of heart sections from mice were stained with WGA to demarcate the cell boundaries (*n *= 6 mice/group). (H) Representative images and quantification of TUNEL staining of heart samples from mice (*n *= 6 mice/group). (I) Representative gel blots and quantification showing levels of cleaved caspase 3 in heart samples from mice (*n *= 6 mice/group). (J) Proposed model of how HINT2 deficiency aggravates DIC. Data are mean ± SEM. *p < 0.05, **p < 0.01, ***p < 0.001. *p* Values are calculated by two‐way ANOVA followed by Tukey's multiple comparisons test.

To investigate whether HINT2 protects against DIC in mice, we overexpressed HINT2 in the myocardium via a single injection of AAV9 carrying *Hint2* under the cTnT promoter. As shown in Figure 8A, myocardial HINT2 protein levels were significantly elevated in mice infected with AAV9–*Hint2*. HINT2 overexpression significantly mitigated DOX‐induced cardiac dysfunction and structural damage, as shown by improved EF, FS, HW/TL (Figure 8B,C,D), and reduced ANP and BNP mRNA levels (Figure 8E,F). WGA staining revealed reduced cardiomyocyte atrophy (Figure 8G). Additionally, Annexin V/PI flow cytometry, TUNEL staining, and cleaved caspase‐3 protein levels indicated lower apoptosis in the HINT2 overexpression group after DOX treatment (Figures S9A and 8H,I). We also examined the impact of HINT2 overexpression on cardiomyocyte contractility. The DOX‐induced reduction in PS and ±dL/dt, along with the prolongation of TP50, were significantly alleviated by HINT2 overexpression (Figure S9B–G). Collectively, these findings confirm that HINT2 overexpression attenuates DIC.

## 3 DISCUSSIONS

HINT2 has been reported to regulate lipid and glucose metabolism and is considered a promising therapeutic target for metabolic diseases.^14^, ^22^, ^23^, ^24^ Previous studies have shown that HINT2 protects heart from ischemia injury. However, the exact role of HINT2 in DIC and its underlying mechanism remained unknown. In this study, we demonstrate for the first time that HINT2 is a key cardioprotective factor in DIC. *Hint2* knockout blocks autolysosome degradation, aggravates cell death and worsens DOX‐induced cardiac dysfunction. In contrast, *Hint2* cardiac‐specific overexpression or NMN treatment protects cardiac function after DOX treatment. Mechanistically, the lack of HINT2 leads to intracellular cholesterol accumulation in cardiomyocytes, inhibiting SREBF2 and subsequently downregulating TFAM. The lack of TFAM disrupts the NAD^+^/NADH balance by impairing OXPHOS complex I expression and activity, leading to lysosomal dysfunction and blocking autophagic flux (Figure 8J). To our knowledge, this is the first report to describe the role of HINT2 in DIC. *Hint2* knockout resulted in cholesterol accumulation in cardiac tissue after DOX treatment, inhibiting SREBF2 and downregulating *TFAM*. To obtain reliable evidence of HINT2's role in DIC, we used both gain‐ and loss‐of‐function models of HINT2 in vivo. We also isolated AMCMs from *Hint2^fl/fl^* mice and model mice to study the effects of HINT2 in vitro, simulating physiological conditions. Finally, we used both acute and chronic DOX injury models in mice to align with clinical cancer treatment practices.

Our previous study highlighted the cGAS–STING pathway as a therapeutic target for endothelial cell dysfunction and mitochondrial disruption in DIC.^25^ Our current findings build on this research, demonstrating how HINT2 influences autophagic processes in cardiomyocytes, offering a new perspective on DIC. We provide the first evidence that HINT2 deficiency worsens cardiac dysfunction by blocking autophagic flux via lysosomal dysfunction. Autophagy, a lysosome‐dependent process, degrades damaged cytoplasmic proteins and organelles to maintain cellular homeostasis.^26^ Autophagy occurs in three forms: macro‐autophagy, micro‐autophagy, and chaperone‐mediated autophagy.^27^ Macro‐autophagy, the most extensively studied form,^27^ was the focus of this study in the context of DIC. The process of macro‐autophagy can be broadly divided into three steps: autophagosome formation, autolysosome biogenesis through autophagosome–lysosome fusion, and lysosome‐dependent autolysosome degradation.^27^ However, the relationship between autophagy and DIC remains unclear. Some studies suggest that DOX triggers excessive autophagy, which is harmful to the heart. Kobayashi et al.^28^ found that DOX induces excessive autophagy flux, causing cardiomyocyte damage, which can be suppressed by activating the cardio‐protective factor GATA4. In contrast, promoting autophagy by inhibiting PI3Kγ improves heart function after DOX treatment, as demonstrated by Li et al.^29^ Similarly, Wang et al.^30^ found that autophagy is activated in the early phase of anthracycline‐induced cardiotoxicity but suppressed in the later phase. They found that autophagy activators reduce DIC when applied in the late phase but not in the early phase. Additionally, Li et al.^13^ found that DOX leads to autolysosome accumulation by inhibiting lysosome acidification. However, most studies have focused on the role of a single organelle in the pathogenesis of anomalous autophagy flux. Here, we propose an organelle interaction model where dysfunction of the OXPHOS chain in mitochondria, due to *Hint2* knockout, induces TFAM deficiency, thereby affecting lysosome acidification through NAD^+^/NADH imbalance. *Hint2*‐specific knockout inhibited lysosomal hydrolysis and acidification after DOX treatment, potentially explaining the decreased lysosomal clearance capacity, which led to excessive autolysosome accumulation in cKO mouse heart tissue following DOX treatment. Previous studies have established that autophagosome or autolysosome accumulation is a key factor inducing excessive ROS production and cardiomyocyte death.^13^, ^31^, ^32^ Our results suggest that autophagy blockage, due to autolysosome accumulation caused by lysosomal dysfunction, is a key mechanism leading to worsened cardiac dysfunction in cKO mice after DOX treatment. This dysfunction is specifically triggered by mitochondrial HINT2 deficiency.

The potential role of mitophagy, a form of autophagy responsible for the selective degradation of mitochondria, is noteworthy in the context of our study. Mitophagy maintains cellular homeostasis and plays a role in several physiological and pathological processes.^33^, ^34^, ^35^ In the heart, mitophagy disruptions have been linked to mitochondrial dysfunction and cardiomyocyte survival, contributing to ischemia/reperfusion injury, cardiomyopathy, and heart failure.^11^, ^36^ DOX‐induced oxidative stress in mitochondria, combined with disruptions in OXPHOS and permeability transition, alters the metabolic and redox circuits of cardiac cells.^11^ These disturbances disrupt autophagy and mitophagy flux, leading to the accumulation of dysfunctional mitochondria and worsening cardiotoxic effects.^37^ In this study on HINT2 and its role in reducing DIC by restoring lysosomal function and promoting autophagy, it is important to note that we did not assess mitophagy. However, given the evidence of mitophagy's role in heart diseases, particularly DIC, future studies should explore this aspect for a more comprehensive understanding of the underlying mechanisms.

The interaction between mitochondria and lysosomes is crucial for maintaining cellular homeostasis and stress response.^38^ Dysfunction in the mitochondrial respiratory chain is closely linked to impaired lysosomal function.^19^, ^20^, ^39^ Fernandez‐Mosquera et al.^20^ found that knocking down the mitochondrial respiratory chain deactivates AMP‐activated protein kinase, reducing lysosomal Ca2^+^ channel mucolipin 1, and ultimately impairing lysosomal function. In our study, we found that TFAM primarily affected mitochondrial complex I following DOX treatment, despite its broader role in regulating mitochondrial gene transcription. This specificity may result from two factors: first, posttranslational modifications like ERK1/2‐mediated phosphorylation can selectively reduce TFAM's binding to mitochondrial DNA, particularly affecting complex I under stress conditions.^40^ Whether HINT2 also influences TFAM through similar modifications warrants further study. Second, complex I genes may be more sensitive to changes in transcription factor levels due to their critical role in the mitochondrial electron transport chain, requiring tighter regulation.^41^ Complex I plays a critical role in regulating the NAD^+^/NADH balance, which is essential for OXPHOS function.^42^ Compared with *Hint2^fl/fl^* mice, AMCMs isolated from cKO mice had a lower NAD^+^/NADH ratio after DOX treatment. Current research shows that NAD^+^ is crucial for maintaining mitochondria–lysosome interactions. For example, Baixauli et al.^19^ discovered that mitochondrial OXPHOS deficiency, leading to a low NAD^+^/NADH ratio, can cause lysosome dysfunction in T cells. The mechanism by which mitochondrial NAD^+^ mediates lysosomal acidification has also been clarified.^43^ Thus, mitochondrial OXPHOS complex I impairment in cKO mice may contribute to lysosomal dysfunction in DIC. It is important to note that NAD^+^ biosynthesis is also associated with enzymes like nicotinamide phosphoribosyl transferase and NMN adenylyl transferases (NMNAT 1–3). NAD+ degradation involves sirtuins 1–7, CD38, and poly(ADP‐ribose) polymerase‐1 (PARP‐1).^44^ However, our data showed no significant difference in the mRNA levels of both NAD^+^ consumption enzymes (e.g., sirtuins 1–7, CD38, PARP‐1) and NAD^+^ synthesis enzymes between *Hint2^fl/fl^* and cKO mice after DOX treatment (Figure S10). This suggests that impaired OXPHOS complex I is likely the main cause of the imbalanced NAD^+^/NADH ratio in cKO mice. In conclusion, *Hint2* knockout may impair mitochondrial complex I, leading to a lower NAD^+^/NADH ratio and consequently, weakened lysosomal function.

NAD^+^/NMN has demonstrated promising therapeutic effects in preclinical models of ischemic heart disease, pressure overload‐induced heart failure, and heart failure with preserved EJ.^45^, ^46^, ^47^ Our study shows that NMN supplementation can mitigate lysosomal dysfunction, enhance lysosomal acidification, improve lysosomal hydrolysis, and reduce autolysosome accumulation in cKO mice after DOX treatment. As previous studies have shown that autolysosome accumulation may lead to cell injury,^13^ our results suggest that improved heart function may partly be due to enhanced clearance of autophagosomes and autolysosomes. Thus, our findings highlight NMN supplementation as a potential therapeutic strategy for lysosomal dysfunction, applicable not only to DIC but also to diseases like glycogen storage disease, Gaucher disease, and Tay‐Sachs disease. Additionally, targeting HINT2 with AAV9 has been shown to protect cardiac function from DIC.

## 4 CONCLUSION

Our study identifies HINT2 as a protective factor against DIC. Mechanistically, *Hint2* knockout impairs OXPHOS complex I, disrupts the NAD^+^/NADH balance, and induces lysosomal dysfunction. This cascade leads to autophagic flux blockage, further exacerbating DIC. Additionally, *Hint2* knockout leads to cholesterol accumulation, downregulating TFAM and decreasing OXPHOS complex I expression. Future clinical studies are required to validate the therapeutic potential of modulating HINT2 in DIC. Our findings suggest that NMN supplementation may restore autophagic flux and alleviate DIC in both cKO and *Hint2^fl/fl^* mice. Further studies are necessary to determine whether NMN supplementation could serve as a clinical strategy to prevent or reduce DIC.

## 5 MATERIALS AND METHODS

### 5.1 Animals

Mice homozygous for the exon‐floxed *Hint2* allele (Cat. No. NM‐CKO‐00086) were generated at the Shanghai Model Organisms Center, Inc. *Hint2^fl/fl^* mice were then crossed with *α‐MHC‐Cre* transgenic mice, which express *Cre* specifically in cardiomyocytes, to obtain cKO mice. *α‐MHC‐cre* mouse lines were described previously.^48^ Tails were genotyped with the following primer sequences: *Hint2* flox: forward 5′‐CAC CTC TGC CTC TGC CCT CT‐3′; and reverse 5′‐TCC CAT TCT CCG TCC AAG C‐3′. *α‐MHC Cre*: forward 5′‐ATG ACA GAC AGA TCC CTC CTA TCT CC‐3′; and reverse 5′‐CTC ATC ACT CGT TGC ATC ATC GAC‐3′.

Mice were euthanized with pentobarbital sodium salt (Sigma; P3761, 125 mg/kg, i.p.), confirmed by unresponsiveness to a firm toe pinch. Anesthesia was induced with isoflurane (4% in 100% O₂ at 0.5 L/min) in a chamber and confirmed by the absence of a toe‐pinch reflex. Anesthesia was maintained using a nose cone during the study.

### 5.2 In vivo experiments

*Hint2^fl/fl^* or cKO mice received a single intraperitoneal injection of DOX (15 mg/kg; Sigma; D1515) or NS as control to induce DIC (Figure S1A). Seven days later, mice were euthanized, and hearts and tibias were collected to calculate HW/TL. Heart samples were analyzed for histological and molecular changes. To test whether NAD+ supplementation improves cardiac function by restoring autophagic flux, mice were treated with NMN (180 mg/kg/day; Selleck; S5259) (Figure S7A).

For myocardium‐specific overexpression of HINT2 or TFAM, 4‐week‐old C57BL/6J mice received a single intravenous injection of AAV9 carrying *Hint2*, TFAM, or mCherry–GFP–LC3 under the cTnT promoter (1 × 10¹¹ viral genomes/mouse) or AAV9–*Con* via the tail vein. These mice were studied after 4 weeks. To enhance clinical relevance, a cumulative DOX dose of 20 mg/kg (5 mg/kg weekly for 4 weeks) was administered (Figure S4A), with echocardiography performed on days 0, 14, and 28.

### 5.3 Transthoracic echocardiography

Cardiac function was assessed in isoflurane‐anesthetized mice using 2D guided M‐mode echocardiography (Visual Sonics Vevo 2100, Canada). EF and FS indices were measured from the parasternal long‐axis view at a heart rate of ∼450 bpm.

### 5.4 Transmission electron microscopy

Left ventricular heart tissues were dissected into small cubes and fixed in 2.5% glutaraldehyde (pH 7.4) for over 2 h. After washing in 0.1 M phosphate buffer, tissues were fixed in 1% osmium tetroxide, dehydrated in graded ethanol, and then in 90% acetone at 4°C. Samples were infiltrated, embedded in Epon Araldite, and sectioned with a diamond knife (Diatome) into ultrathin sections. Sections were poststained with 2% uranyl acetate and lead citrate, and images were captured using a CM‐120 transmission electron microscope (Philips, The Netherlands).

### 5.5 hiPSC‐CM, NMVM, AMCM isolation, culture, DOX treatment, immunofluorescence staining, live/death staining, Annexin V/PI flow cytometry, adenoviral infection, and contractile ability measurement

Commercially purchased hiPSC‐CMs (LingYin0102012; Ronovation, Hangzhou, China) were cultured for 10 days before experiments, following the company's guidelines. Experiments proceeded after confirming continuous and uniform cell contraction under a microscope.

NMVMs were isolated from C57BL/6J mice (1–3 days old) using a modified enzymatic digestion method as previously described.^49^

The isolation of AMCMs was performed as described previously.^50^ The descending aorta and inferior vena cava were cut, and EDTA buffer was injected into the right ventricle. The heart was excised, and EDTA buffer was injected into the left ventricle near the apex. Subsequently, 3 mL perfusion buffer, digestion buffer, and 40 mL collagenase buffer were sequentially injected into the apex. After digestion, the left ventricle was minced into ∼1 mm^3^ pieces and triturated. Pure cardiomyocytes were isolated using gravity settling and used for further studies.

To investigate DIC, hiPSC‐CMs, NMVMs, and AMCMs were treated with 1 µM DOX for the indicated times. HINT2 protein expression was measured via western blot or immunofluorescence staining (ThermoFisher PA5‐54286). AMCM viability was assessed using the Calcein/PI Cell Viability/Cytotoxicity Assay Kit (Beyotime; C2015S), and apoptosis was analyzed by Annexin V/PI flow cytometry (Yeasen; 40302ES50) on a CytoFLEX/DxFLEX flow cytometer (Beckman Coulter). Data were quantified with FlowJo X 10.0.7 software (Tree Star). To evaluate NAD+ protection, NMN (1 mM) was added to AMCMs for 4 h.

For adenoviral transfection, AMCMs were incubated with *Hint2* adenovirus for 12 h, harvested for Western blot validation, or used for further experiments at 48 h. Autophagic flux was assessed by infecting AMCMs with mCherry–GFP–LC3 adenovirus (HANBIO; MOI 20) for 24 h before additional treatment. Cardiomyocyte mechanical properties were measured using the SoftEdge MyoCam system (IonOptix) with cells superfused in BDM‐free culture medium and calcium buffer (130 mM NaCl, 5.4 mM KCl, 10 mM HEPES, 1.8 mM CaCl_2_, 0.5 mM MgCl_2_, 10 mM glucose, pH 7.4). Stimulation was applied at 0.5 Hz using platinum wires connected to an FHC stimulator (Brunswick, NE, USA).

### 5.6 Immunoblotting

Total protein was extracted from left ventricular tissue or cultured cells using RIPA lysis buffer (Beyotime; P0013B) with protease and phosphatase inhibitor cocktails (Beyotime; P1010 and P1081) and PMSF (Beyotime; ST506). Protein concentration was measured with the BCA Protein Assay Kit (Beyotime; P0012S). Equal protein amounts (10–15 µg) were separated by electrophoresis, transferred to PVDF membranes, blocked with 5% BSA, and incubated with primary antibodies at 4°C overnight, followed by secondary antibodies for 1 h at room temperature. Membranes were scanned using Chemiluminescence (Luminata™ Forte, Millipore) and the ChemiDoc™ Imaging System (Bio‐Rad, CA, USA).

### 5.7 RNA isolation and polymerase chain reaction analysis

Total RNA was extracted from heart tissue or cultured cells using Trizol™ Reagent (Invitrogen; 15596‐026). RNA concentration and purity were measured with Nanodrop, and 1000 ng of RNA was reverse transcribed into cDNA using the PrimeScript™ Reverse Transcription Master (TaKaRa; RR036A). Quantitative PCR was performed in a 20 µL reaction system (0.8 µL cDNA, 5 µL SYBR, 0.4 µL primers, 3.8 µL ddH_2_O) under the following conditions: 30 s at 95°C, followed by 40 cycles of 5 s at 95°C and 30 s at 60°C. Fluorescent signals were normalized to GAPDH using the 2−ΔΔCT method.

### 5.8 Lysosomal PH measurement

Lysosomal pH was measured using LysoSensor Yellow/Blue DND‐160 (ThermoFisher; L7545) per the manufacturer's instructions. AMCMs from *Hint2^fl/fl^* or cKO mice were plated in 96‐well plates, treated with DOX (1 µM, 4 h) or PBS, washed, and incubated with 5 µM LysoSensor at 37°C for 5 min. Emission at 535 nm was recorded at excitation wavelengths of 340 and 380 nm to calculate the 340ex/380ex ratio. A lower ratio indicates a lower pH^51^. Representative images were obtained using a confocal microscope.

### 5.9 TUNEL and WGA assay, CTSB, CTSD, and OXPHOS complex Ι enzyme activity assay, total cholesterol assay, mitochondria labeling

The One Step TUNEL Apoptosis Assay Kit (Beyotime; C1090) was used to detect apoptotic cells in formalin‐fixed heart tissue following the manufacturer's instructions. Cardiomyocyte cross‐sectional areas were stained with WGA (Invitrogen; W21405), and nuclei were stained with DAPI (Beyotime; C1002). Fluorescence microscopy (Olympus, Japan) was used for imaging.

CTSB and CTSD enzymatic activities were measured using the CTSB Activity Assay Kit (Abcam; ab65300) and CTSD Activity Assay Kit (Abcam; ab65302), respectively, with fluorometry at Ex/Em = 400/505 nm and 328/460 nm. Heart tissues from DOX‐treated mice were prepared according to the kits’ instructions.

Mitochondria were isolated from heart samples using a tissue mitochondria isolation kit (Beyotime; C3606), and complex I activity was assessed using the Complex I Enzyme Activity Assay Kit (Abcam; ab109721), normalized to the control group. Cholesterol levels were measured from 10 mg homogenized tissue using the Cholesterol/Cholesterol Ester Quantification Kit (Abcam; ab65359).

Mitochondrial morphology was evaluated with MitoTracker™ Dyes (ThermoFisher; M22426) incubated at 37°C for 30 min. Confocal microscopy (SP8; Leica Microsystems, USA) was used for imaging, and Fiji software's Mitochondria Analyzer quantified structural parameters.

### 5.10 Mass spectrometry

Mouse heart tissues were shredded in liquid nitrogen and lysed in ice‐cold protein extraction buffer (7 M urea, 2 M thiourea, 0.1% PMSF, 65 mM DTT) for 30 min. The lysate was centrifuged at 16000 *g* for 15 min, and protein concentration in the supernatant was measured via Bradford assay. A total of 200 µg protein was reduced with 10 mM DTT at 37°C for 60 min, alkylated with 20 mM iodoacetamide at room temperature for 30 min in the dark, and precipitated in ice‐cold acetone overnight at −80°C. The precipitation of samples was dissolved in 200 µL of 50 mM ammonium bicarbonate, digested with trypsin (1:50 ratio) at 37°C overnight, desalted with a SepPak tC18 cartridge (Waters), and subjected to high‐pH two‐dimensional separation. Samples were separated on an analytical column (BEH C18; Waters) under a 20‐min linear gradient (5–45% buffer B) at 300 nL/min, lyophilized, and resuspended in 0.1% formic acid. Further separation was performed using the nanoElute® system (Bruker Daltonik) with an analytical column (ThermoFisher) under a 60‐min gradient. Peptides were analyzed using a TIMS–TOF Pro mass spectrometer under specified parameters. Peptide identification employed Swissprot with an FDR *q*‐value < 0.05, while differentially expressed proteins were identified using Student's *t*‐test (*p* < 0.05) and fold‐change >1.5 or <1/1.5.

### 5.11 GSE42177 analysis

Gene expression analysis was reported by Jain et al. (https://www.ncbi.nlm.nih.gov/geo/query/acc.cgi?acc=GSE42177). Six samples (GSM1034369–GSM1034371 as controls, GSM1034375–GSM1034377 as DOX‐treated) were analyzed using GEO2R (*p* < 0.05, fold‐change >1.25 or <1/1.25).

### 5.12 NAD^+^/NADH measurement

Total NAD^+^/NADH from heart tissue or AMCM was extracted by the NAD^+^/NADH detection kit (Beyotime; S0175). The NADH content was detected after NAD^+^ was decomposed at 60°C for 30 min.

### 5.13 Chromatin immunoprecipitation

Chromatin immunoprecipitation was performed using the SimpleChIP^®^ Plus Sonication Chromatin IP Kit (Cell Signaling Technology; 56383) following the manufacturer's protocol. Cells were fixed with 1% formaldehyde for 10 min at room temperature, neutralized with glycine, washed with cold PBS, and lysed in ChIP Sonication Cell Lysis Buffer with protease inhibitors. Lysates were sonicated on ice (16 min, 1 s on/1 s off cycle) and centrifuged. The supernatant was immunoprecipitated overnight at 4°C with anti‐Flag antibody or IgG (control), followed by 2 h incubation with Protein G Magnetic beads at 4°C. After elution and cross‐link reversal, DNA was isolated for PCR analysis.

### 5.14 Analysis of *TFAM* promoter activity

SREBF2 binding sites on the *TFAM* promoter were identified via CHIP assay. The *TFAM* promoter and SREBF2 sequences were inserted upstream of the pGL3‐Basic luciferase reporter plasmid, and SREBF2 was overexpressed using PCDNA3.1 (Hanbio Biotechnology, Shanghai). HEK293T cells were co‐transfected with Lipofectamine 3000 (Invitrogen) following the manufacturer's protocol. After 48 h of transfection and subsequent treatment, firefly (Fluc) and Renilla (Rluc) luciferase activities were measured using the Dual‐Luciferase Reporter Assay system (E1910; Promega).

### 5.15 Statistical analyses

All values were analyzed using Prism 8.0 (GraphPad Software, Inc., La Jolla, CA). Continuous variables were expressed as mean ± SEM. Normal distribution was assessed by the Shapiro–Wilk test. Differences in normal data were tested using the Student's *t*‐test (two groups) or one‐way ANOVA (≥3 groups) with Tukey's post hoc test. Mann–Whitney *U* or Kruskal–Wallis H tests were applied for nonnormal data. Statistical significance was set at ∗*p* ≤ 0.05, ∗∗*p* ≤ 0.01, ∗∗∗*p* ≤ 0.001, and ∗∗∗∗*p* ≤ 0.0001.

## AUTHOR CONTRIBUTIONS

*Research design*: Hao Jiang, Jinyan Zhang, Kai Hu, Aijun Sun, and Junbo Ge. *Experimental operations*: Hao Jiang, Jinyan Zhang, Daile Jia, Liwei Liu, Jinfeng Gao, Suling Ding, Luna He, and Yiqin Shi. *Guidance on animal experiments*: Zhen Dong and Xiaolei Sun. *Mass spectrometry data availability*: Jinyan Zhang and Daile Jia. *Results analysis and visualization*: Jinyan Zhang. *Writing*: Hao Jiang, Jinyan Zhang, Beijian Zhang,Wenlong Yang, and Tiantong Ou. All authors have read and approved the final manuscript.

## CONFLICT OF INTEREST STATEMENT

The authors declare no conflicts of interest.

### ETHICS STATEMENT

All animal experimental procedures were in compliance with the Guide for the Care and Use of Laboratory Animals, published by the US National Institutes of Health (NIH publication no. 85–23, revised 1996), and were reviewed and approved by the animal ethics committee at Zhongshan Hospital, Fudan University, China (IACUC approval number: 2020–039).

## Supporting information



Supporting Information

## Data Availability

Data generated and described in this article can be obtained from the correspoding author upon resonable request.
