# Supplementary material for: Histidine triad nucleotide‐binding protein 2 attenuates doxorubicin‐induced cardiotoxicity through restoring lysosomal function and promoting autophagy in mice
Source: MedComm (2020). 2025 Feb 17;6(3):e70075. doi: 10.1002/mco2.70075 (PMC11831189; doi:10.1002/mco2.70075)
Supplement: Supplementary file 1 — Supporting Information [file MCO2-6-e70075-s001.docx]

**Histidine triad nucleotide-binding protein 2 attenuates doxorubicin-induced cardiotoxicity through restoring lysosomal function and promoting autophagy in mice**

Hao Jiang^1,2,3,4,5#^, Jinyan Zhang^1,3,4,5#^, Daile Jia^1,2,3,4,5#^, Liwei Liu^1,3,4,5#^, Jinfeng Gao^1,3,4,5^, Beijian Zhang^1,2,3,4,5^, Zhen Dong^1,3,4,5^, Xiaolei Sun^1,3,4,5^, Wenlong Yang^1,2,3,4,5^, Tiantong Ou^1,3,4,5^, Suling Ding^1,3,4,5^, Luna He^7^,Yiqin Shi^7^, Kai Hu^1,3,4,5^, Aijun Sun^1,2,3,4,5,6*^, Junbo Ge^1,2,3,4,5,6*^

1. Department of Cardiology, Zhongshan Hospital, Fudan University, Shanghai Institute of Cardiovascular Diseases, China.

2. National Clinical Research Center for Interventional Medicine, Shanghai, China.

3. State Key Laboratory of Cardiovascular Diseases, Zhongshan Hospital, Fudan University.

4. NHC Key Laboratory of Ischemic Heart Diseases.

5. Key Laboratory of Viral Heart Diseases, Chinese Academy of Medical Sciences, Shanghai, China.

6. Institutes of Biomedical Sciences, Fudan University, Shanghai, China

7. Department of Nephrology, Zhongshan Hospital, Fudan University, Shanghai, China

*Correspondence to:

Junbo Ge, MD, Department of Cardiology, Zhongshan Hospital, Fudan University. Shanghai Institute of Cardiovascular Diseases. No. 1609 Xietu Road, District Xuhui, Shanghai, 200025; China; E-mail: [jbge@zs-hospital.sh.cn](mailto:jbge@zs-hospital.sh.cn)

Aijun Sun, MD, Department of Cardiology, Zhongshan Hospital, Fudan University. Shanghai Institute of Cardiovascular Diseases. No. 1609 Xietu Road, District Xuhui, Shanghai, 200025; China; E-mail: [sun.aijun@zs-hospital.sh.cn](mailto:sun.aijun@zs-hospital.sh.cn)

# Hao Jiang, Jinyan Zhang, Daile Jia and Liwei Liu contributed equally to this article.

**
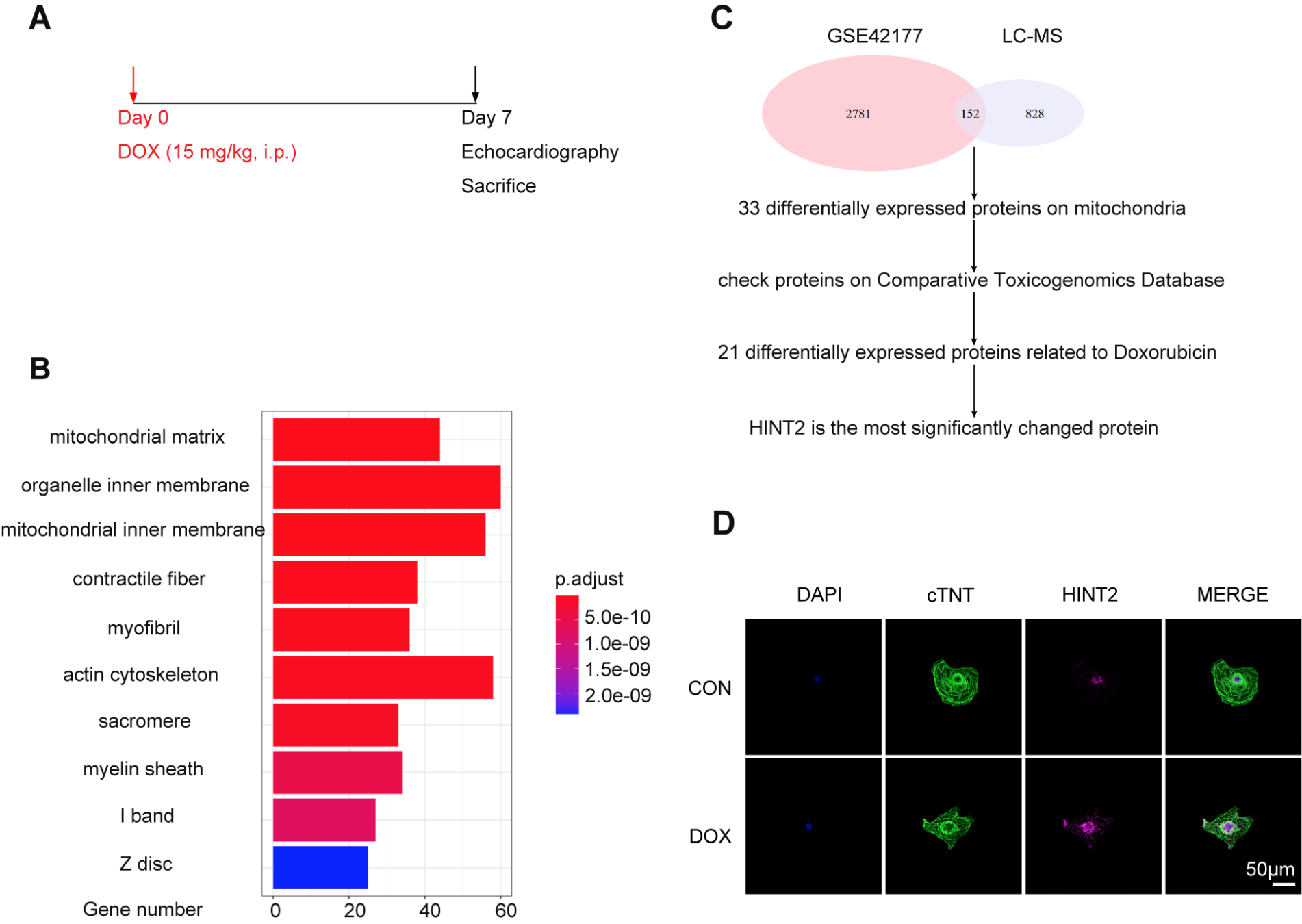
**

**Figure S1. Related processes of mass spectrometry and exploration of autophagy-related proteins.**

**a** Experimental scheme of acute DIC model. **b, c** GO analysis of differentially expressed protein in heart samples from mice treated with NS or DOX (7 days after 15mg/kg DOX,i.p., single, *n*=3 mice/group). **d** Representative images of HINT2 immunofluorescent analyses of hiPSC-CMs after DOX treatment (1uM, 4hours).


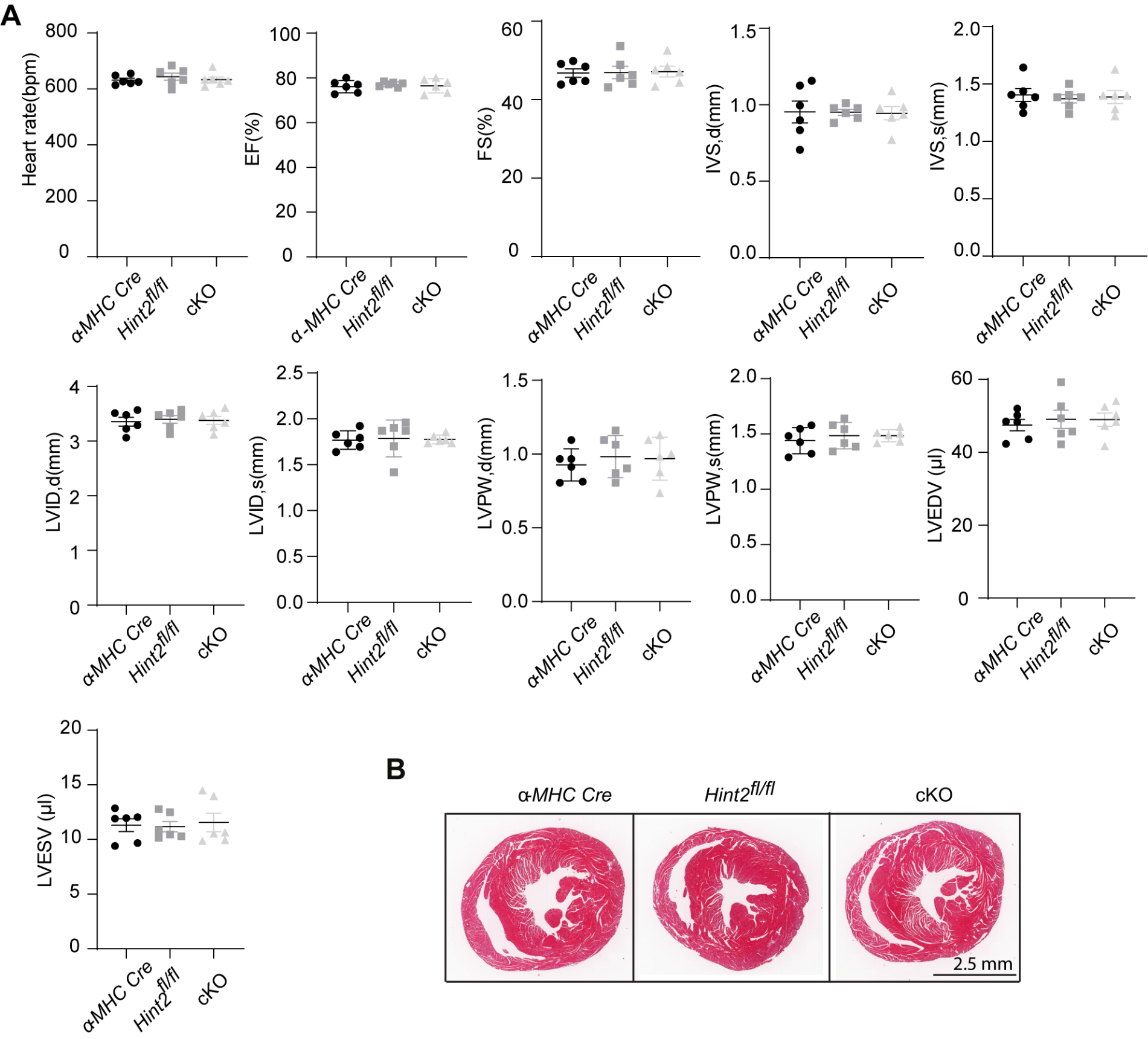


**Figure S2. Baseline echocardiography parameters and histology.**

**a** Baseline echocardiography parameters of mice at age of 8 weeks (*n*=6 mice/group). b Representative images from Hematoxylin and Eosin (HE) stained heart sections from mice. P values are calculated by two-way ANOVA followed by Tukey’s multiple comparisons test.


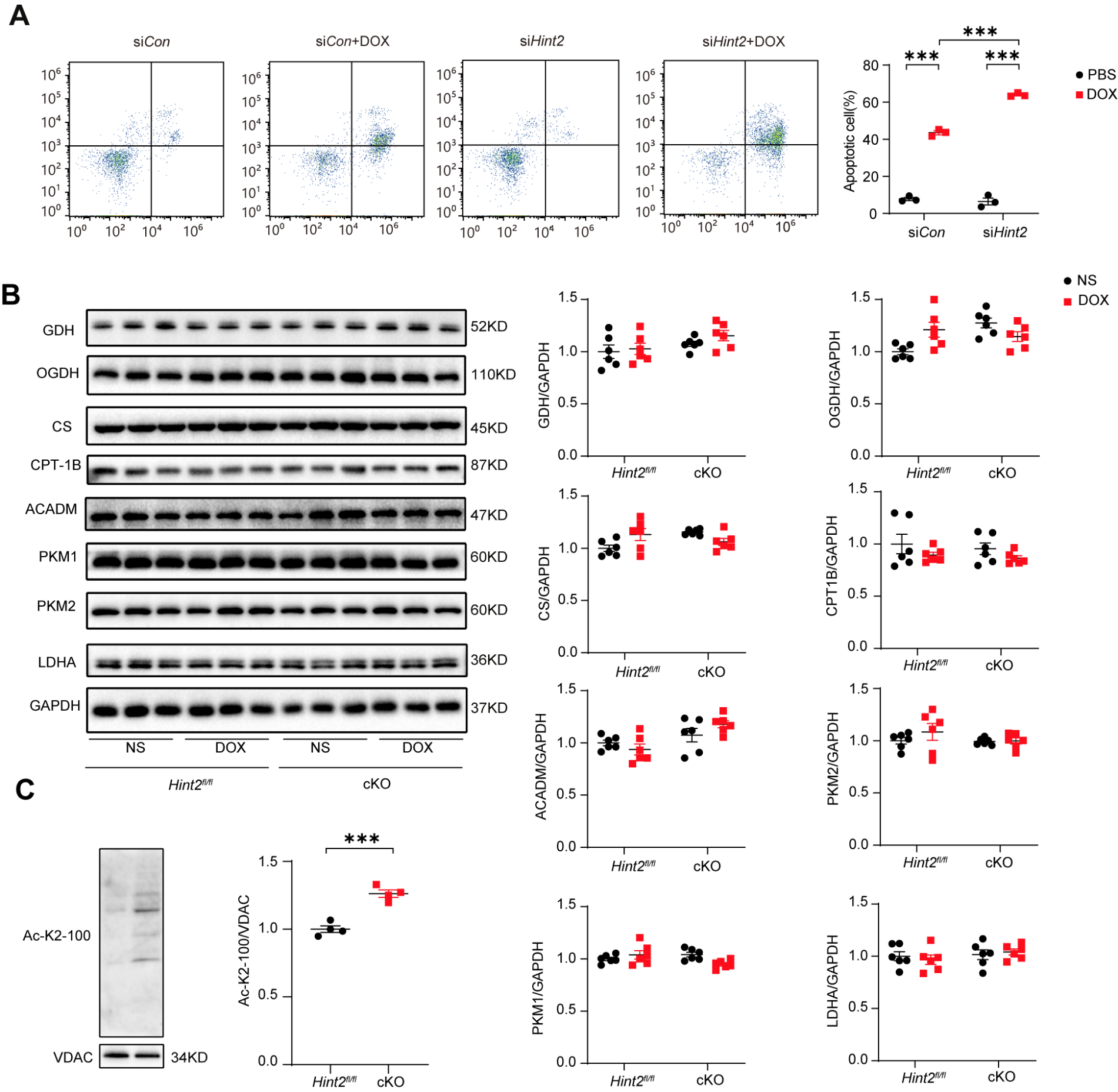


**Figure S3. Apoptosis, metabolic analysis and mitochondria protein acetylation in mice myocardium.**

**a** Annexin V/PI flow cytometry analysis of NMVMs (*n*= 3 samples/group)**. b, c** Representative gel blots and quantification showing levels of GDH, OGDH, CS, CPT-1B, ACADm, PKM2, PKM1, LDHA (*n*= 6 mice/group) and levels of mitochondria protein acetylation in heart tissues (*n*=4 mice/group) (7 days after 15mg/kg DOX,i.p. , single injection,). Data are mean ± SEM. *P < 0.05, **P < 0.01, ***P < 0.001. P values are calculated by unpaired Student’s t test or two-way ANOVA followed by Tukey’s multiple comparisons test.

**
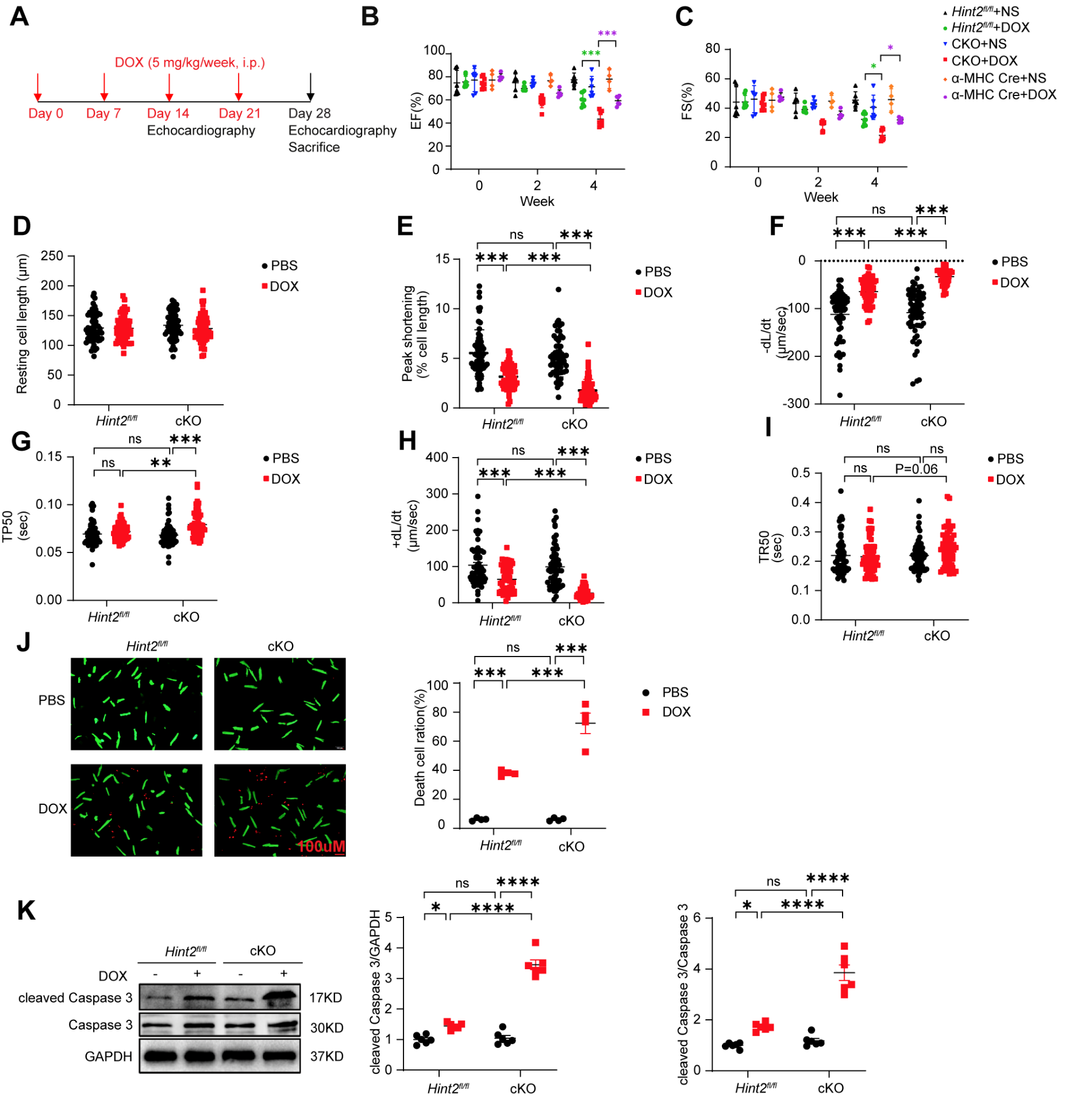
**

**Figure S4. HINT2 deficiency worsens the function of AMCMs isolated from *Hint2^fl/fl^* and cKO mice after DOX treatment and exacerbate cardiac dysfunction in chronic DOX -induced cardiotoxicity model.**

**a** Experimental scheme of chronic DOX -induced cardiotoxicity model. **b, c** Quantification of EF and FS of mice at 14, 28 days after chronic DOX treatment (*n*=4-8 mice/group). **d** Resting cell length. **e** Peak shortening (PS, normalized to resting cell length). **f** Maximal velocity of shortening (− dL/dt). **g** Time-to-50% PS (TP50). **h** Maximal velocity of relengthening (+ dL/dt). **i** Time-to-50% relengthening (TR50). After PBS or DOX treatment (1uM, 4hours). *n*=60-61 cells from 3 mice per group. **j** Representative images and quantification of live (green)/dead (red) viability of AMCMs (1uM, 4hours) (*n*=4 wells/group). **k** Representative gel blots and quantification showing levels of cleaved caspase-3 of AMCMs (1uM, 4hours) (*n*=6 wells/group). Data are mean ± SEM. *P < 0.05, **P < 0.01, ***P < 0.001, ****P < 0.0001. P values are calculated by two-way ANOVA followed by Tukey’s multiple comparisons test.

**
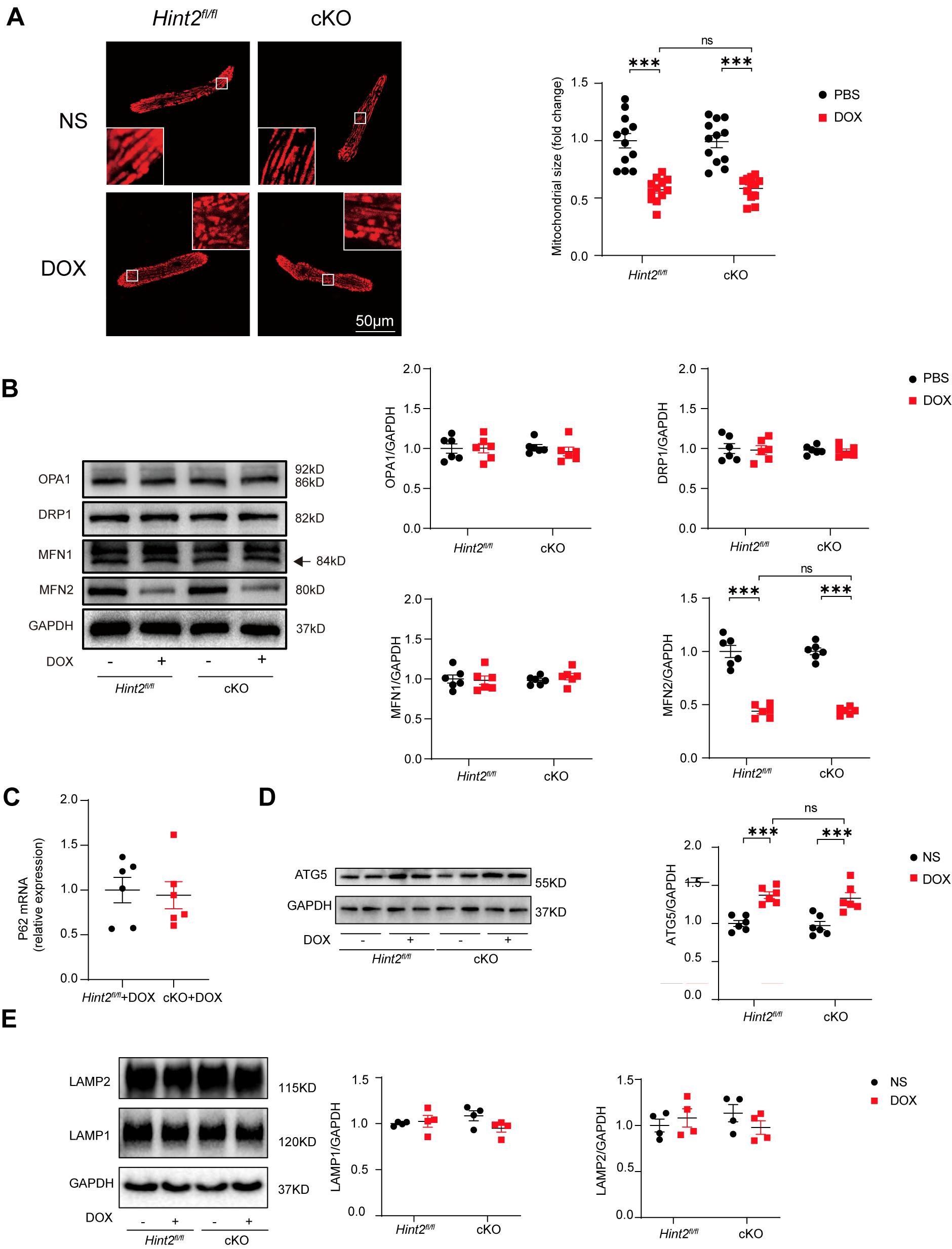
Figure S5. Mitochondrial size of AMCMs and related protein expression and mRNA expression of P62.**

**a** MitoTracker™ Dyes for visualization of mitochondrial size of AMCMs (*n*=12 samples/group). **b, d, e** Representative gel blots and quantification showing levels of Opa1, Drp1, Mfn1, Mfn2, and ATG5 (*n*=6 mice/group), LAMP1/2 (*n*=4 mice/group) in heart tissues from mice (7 days after 15mg/kg DOX,i.p. , single injection). **c** mRNA expression of p62 in heart tissues from mice (7 days after 15mg/kg DOX,i.p. , single injection, *n*=6 mice/group). Data are mean ± SEM. *P < 0.05, **P < 0.01, ***P < 0.001. P values are calculated by unpaired Student’s t test or two-way ANOVA followed by Tukey’s multiple comparisons test.


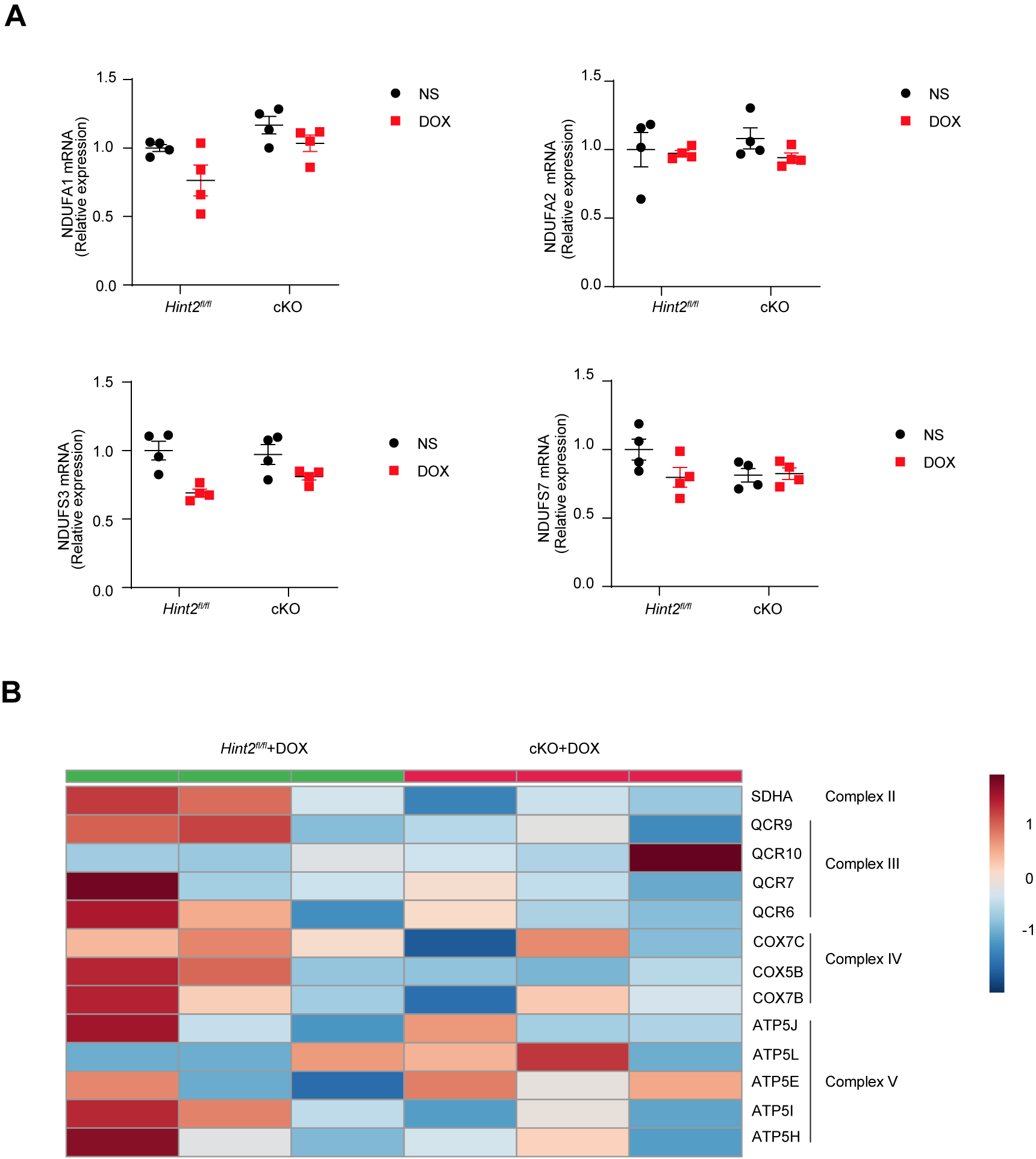


**Figure S6. HINT2 deficiency did not influence nuclear-encoded subunits of Complex I and subunits of Complex II, III, IV, V.**

**a** mRNA expression of nulcear-encoded subunits NDUFA1, NDUFA2, NDUFS3 and NDUFS7 of Complex I in mice heart tissues (7 days after 15mg/kg DOX,i.p. , single injection, *n*=4 mice/group). **b** Heatmap demonstrating mean normalized protein expression of subunits of Complex II, III, IV, V in myocardium (7 days after 15mg/kg DOX,i.p. , single injection, *n*=3 mice/group). Data are mean ± SEM. P values are calculated by two-way ANOVA followed by Tukey’s multiple comparisons test.


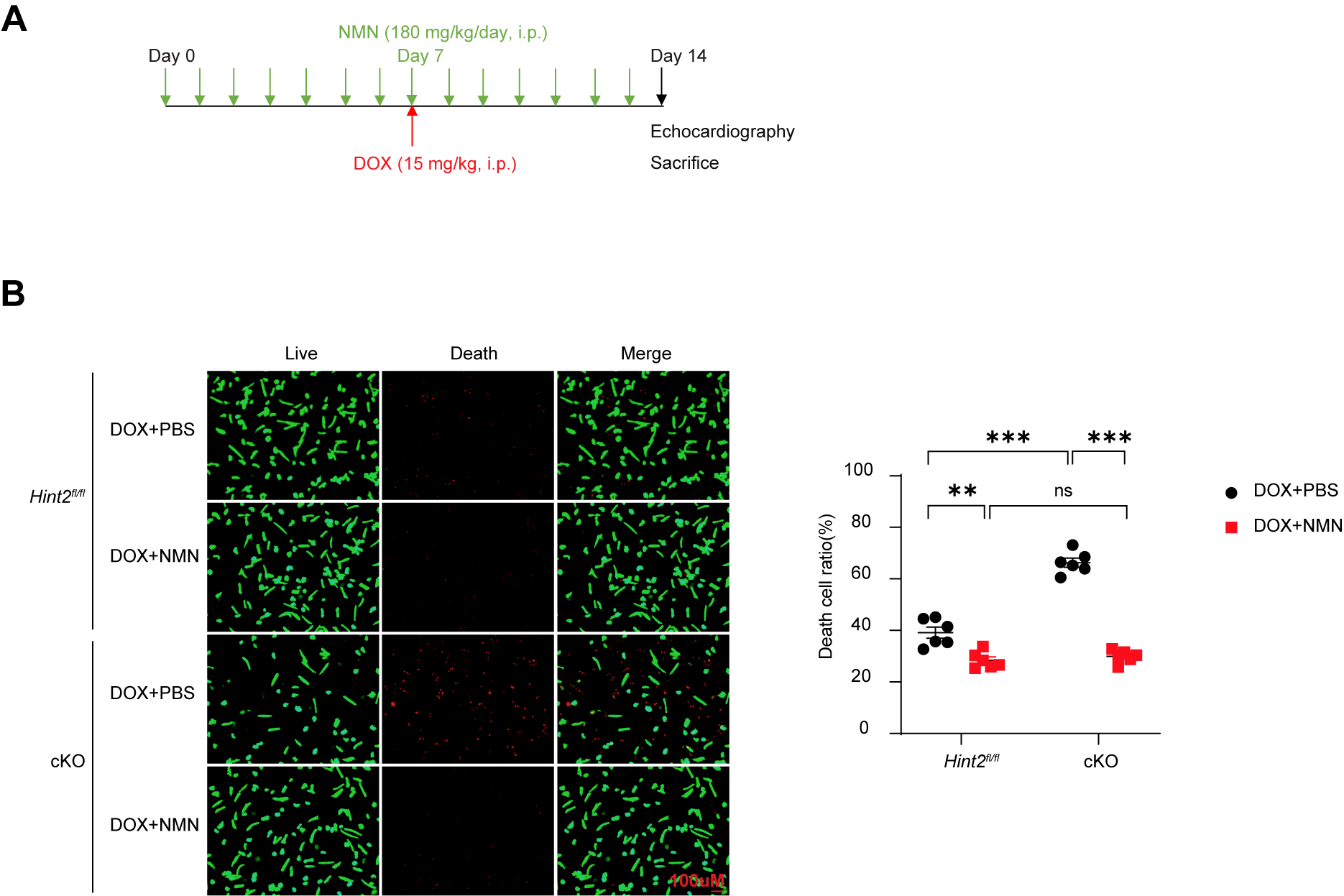


**Figure S7. NMN alleviate AMCMs death induced by DOX in vitro and NMN replenishment design for animal**

**a** Experimental scheme of NMN replenishment for mouse model of acute DIC. **b** Representative images and quantification of live/dead viability of AMCMs after PBS or DOX treatment (1uM, 4hours) with or without NMN replenishment (*n*=6 wells/group). Data are mean ± SEM. *P < 0.05, **P < 0.01, ***P < 0.001. P values are calculated by two-way ANOVA followed by Tukey’s multiple comparisons test.

**
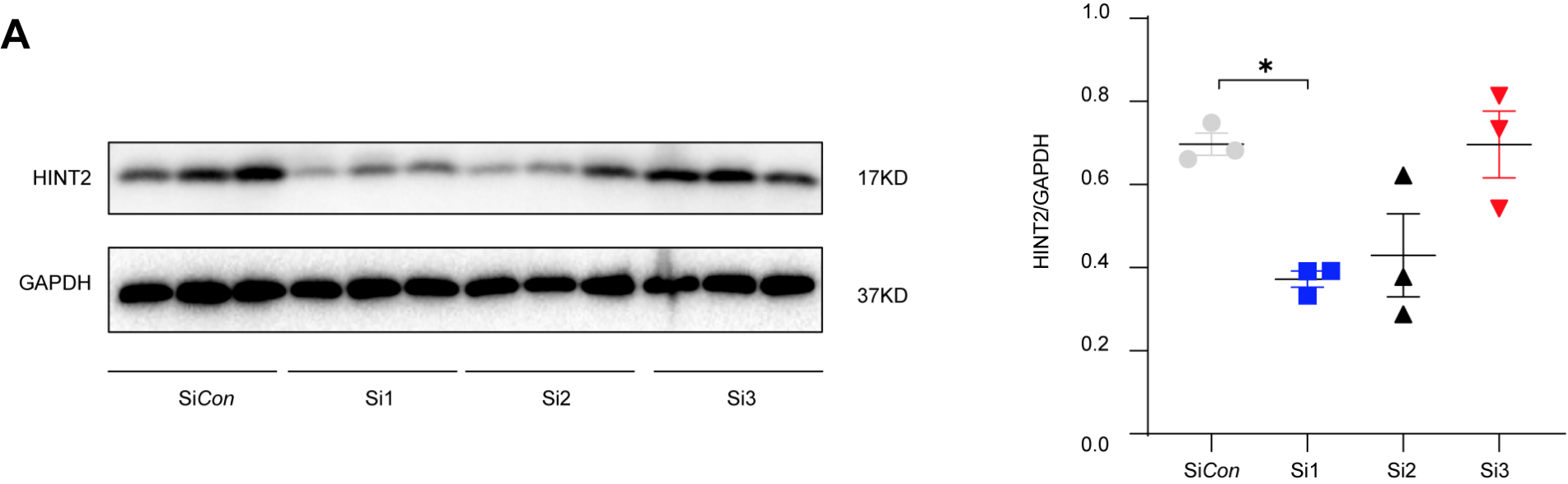
**

**Figure S8. Knockdown of *Hint2* in HEK293T cells.**

**a** Representative gel blots and quantification showing levels of HINT2 in HEK293T transfected with indicated siRNA (*n*=3 wells/group). Data are mean ± SEM. *P < 0.05, **P < 0.01, ***P < 0.001 P values are calculated by two-way ANOVA followed by Tukey’s multiple comparisons test.


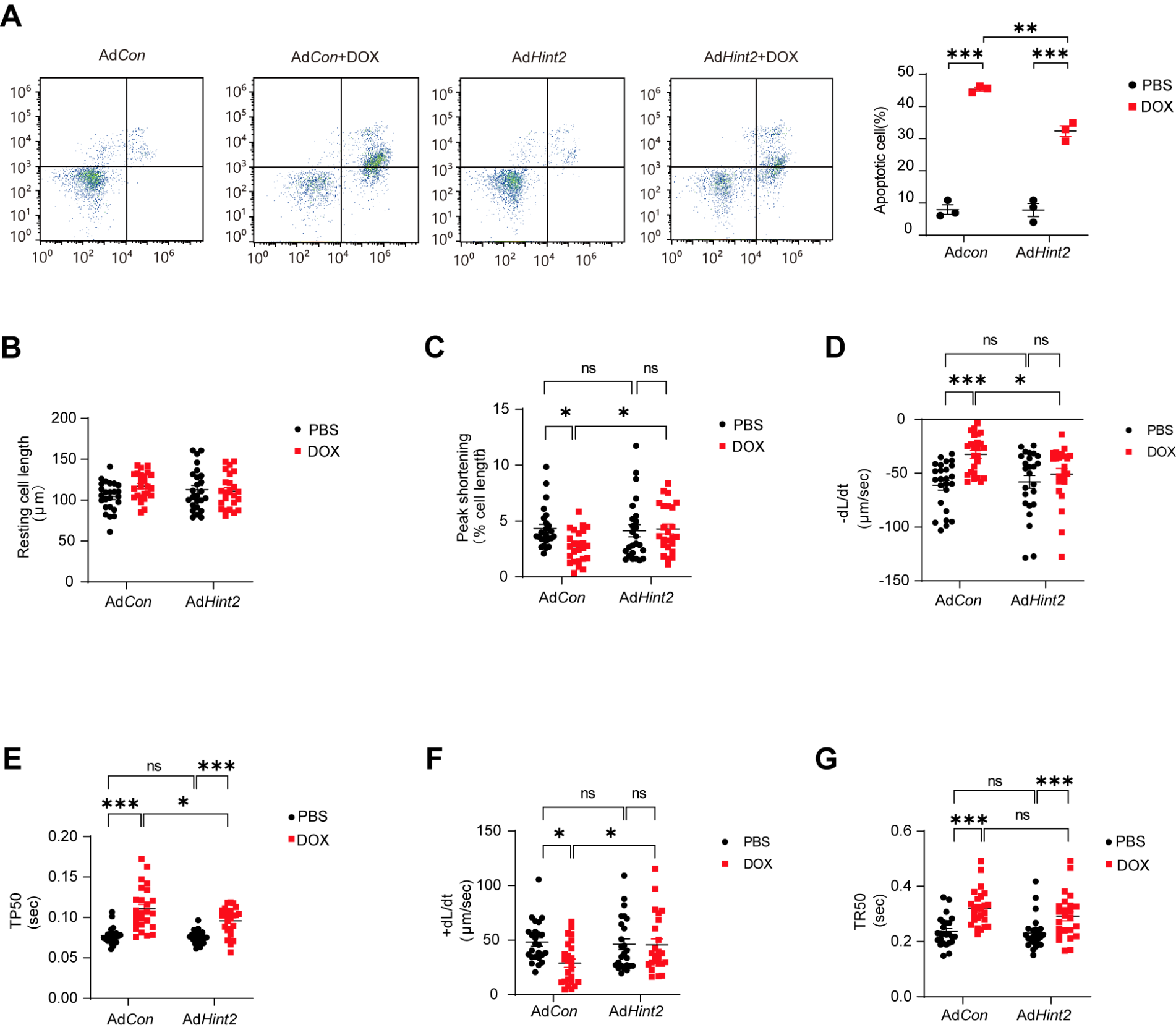


**Figure S9. HINT2 overexpression improve apoptosis and contractile function of isolated cardiomyocytes.**

**a** Annexin V/PI flow cytometry analysis of NMVMs (*n*= 3 samples/group)**. b** Resting cell length. **c** PS. **d** − dL/dt. **e** PS (TP50). **f** + dL/dt. **g** TR50. After PBS or DOX treatment (1uM, 4hours) (*n*=24-25 cells from 3 mice per group). Data are mean ± SEM. *P < 0.05, **P < 0.01, ***P < 0.001. P values are calculated by two-way ANOVA followed by Tukey’s multiple comparisons test.

**
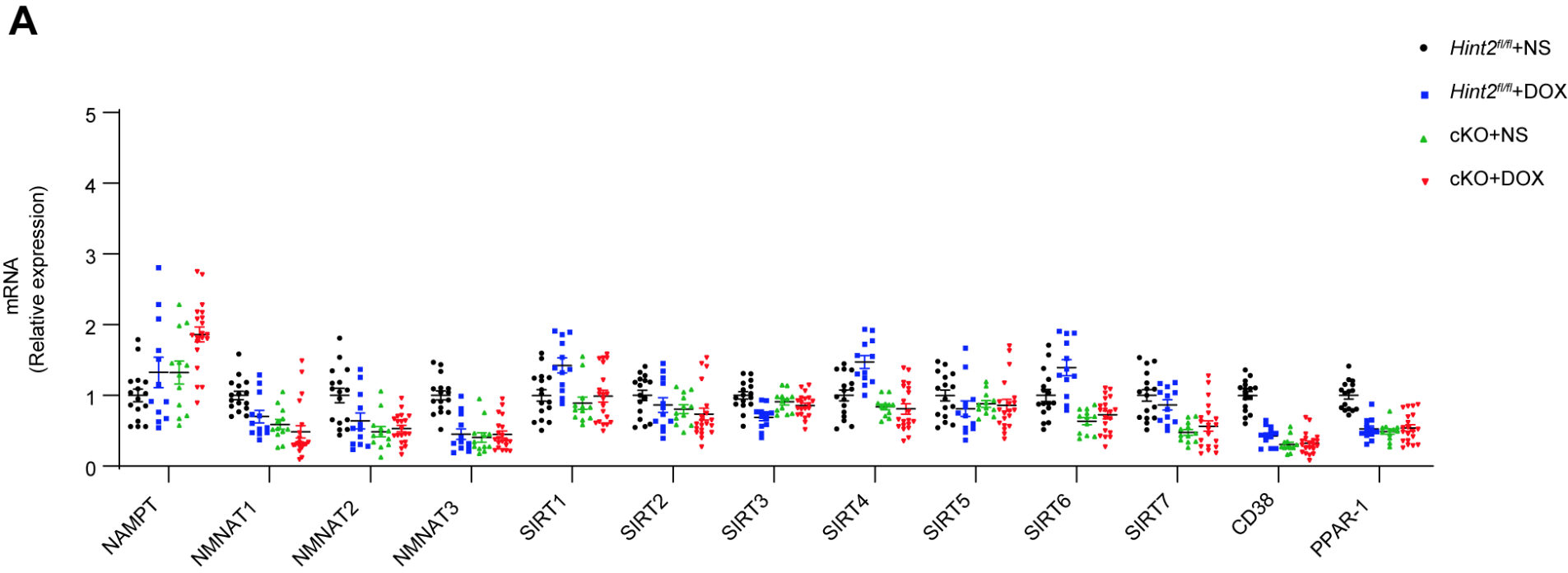
Figure S10. RT-PCR analysis of NAD^+^ metabolism enzymes from myocardium.**

**a** mRNA expression of NAD^+^ metabolism enzyme in *Hint2^fl/fl^*and cKO mice heart samples at 7 days after NS or DOX treatment (*n*=12~20 mice/group). Data are mean ± SEM. P values are calculated by two-way ANOVA followed by Tukey’s multiple comparisons test.

| **Target name** | **Forward sequence** | **Reverse sequence** |
| --- | --- | --- |
| **ANP** | GCTTCCAGGCCATATTGGAG | GGGGGCATGACCTCATCTT |
| **BNP** | GAGGTCACTCCTATCCTCTGG | GCCATTTCCTCCGACTTTTCTC |
| **GAPDH** | AGGTCGGTGTGAACGGATTTG | TGTAGACCATGTAGTTGAGGTCA |
| **ND1** | CTCAACCTAGCAGAAACAAACC | GGCCGGCTGCGTATTCTAC |
| **ND6** | TTGGGAGATTGGTTGATGTAT | TGCCGCTACCCCAATCC |
| **SIRT1** | CTCCTTGGAGACTGCGATGTT | AGGTGTTGGTGGCAACTCTG |
| **SIRT2** | CCTCTAACCACCCAGGCAAG | TAGGGGTTTTGGGGTAGCCT |
| **SIRT3** | TCACAACCCCAAGCCCTTTT | GTGGGCTTCAACCAGCTTTG |
| **SIRT4** | AAAGGTGGGACTTTACGCCC | TGAGTCACCAACCAGTGCAG |
| **SIRT5** | ACTTCTTAACCGCCCTGTGG | TTGGGGCTTGAAGGGTGTTT |
| **SIRT6** | ATCTTCGACCCACCAGAGGAG | TGGAGGACTGCCACATTAGC |
| **SIRT7** | GTGACGGTCGTCTTTAGCCA | GAGGTAAGGCAGTGGTGGAC |
| **NMNAT1** | CAACATCGCTGTCAGAGGGT | TAAAAGAGCCACAGGCCAGG |
| **NMNAT2** | AAGATCTTGGGAAAGGTGGGAG | TGAAGCGCTCCACTGGT |
| **NMNAT3** | AAGGTCAAGTTCACTCGCGG | CCGCAGATCGCCCTAAGAC |
| **NAMPT** | TGGGGTGAAGACCTGAGACA | TGGCAGCAACTTGTAGCCTT |
| **PARP1** | GGCAGCCTGATGTTGAGGT | GCGTACTCCGCTAAAAAGTCAC |
| **CD38** | TCTCTAGGAAAGCCCAGATCG | GTCCACACCAGGAGTGAGC |
| **Mouse 18s** | CGCGGTTCTATTTTGTTGGT | AGTCGGCATCGTTTATGGTC |
| **Human 18s** | GGACCAGAGCGAAAGCATTTGCC | TCAATCTCGGGTGGCTGAACGC |
| **Human Hint2** | CAGCCCCAACCATCTTCTCC | GCCTGGCTAATCCGAGGAATG |
| **NDUFA1** | CCGGAAGAGAGGTAAAGCCG | ACATCTCCGCACCGTTACTC |
| **NDUFA2** | CGCTGTACAGTGTCCCTTCA | CAGTGTTGCGCAGTAAGAGG |
| **NDUFS3** | ATCCTTGGCTGACTTGACGG | CGCAGAGACAGCAGGTTGTA |
| **NDUFS7** | TGCGCAGAGTTCATCAGAGT | ATGAGAGAGCTTGGGGACCA |
| **TFAM** | GGAATGTGGAGCGTGCTAAAA | ACAAGACTGATAGACGAGGGG |

**Table S1. Primer sequences for related experiments**

| **Antibody** | **Item No.** | **Applications** |
| --- | --- | --- |
| **HINT2** | ThermoFisher PA5-50226 | WB (1:1000) |
| **HINT2** | ThermoFisher PA5-54286 | IF (1:100) |
| **Anti-Cardiac Troponin T** | Abcam ab8295 | IF (1:200) |
| **Caspase 3** | proteintech 19677-1-AP | WB (1:1000) |
| **LC3A/B** | CST 12741S | WB (1:1000) |
| **Anti-SQSTM1 / p62** | Abcam ab56416 | WB (1:1000) |
| **Cathepsin D** | Abcam ab75852 | WB (1:1000) |
| **Cathepsin B** | Abcam ab214428 | WB (1:1000) |
| **OXPHOS** | Abcam ab110413 | WB (1:5000) |
| **VDAC** | CST 4866S | WB (1:1000) |
| **Anti-mtTFA** | Abcam ab307302 | WB (1:1000) |
| **SREBF2** | Santa Cruz | WB (1:500), IF (1:50) |
| **GAPDH** | CST 2118S | WB (1:1000) |
| **GDH** | CST 4060S | WB (1:1000) |
| **OGDH** | CST 26865S | WB (1:1000) |
| **Citrate Synthase** | CST 14309S | WB (1:1000) |
| **CPT1B** | CST 41803S | WB (1:1000) |
| **ACADM** | GeneTex GTX101393 | WB (1:1000) |
| **PKM1** | CST 7067S | WB (1:1000) |
| **PKM2** | CST 4053S | WB (1:1000) |
| **LDHA** | Abclonal A21893 | WB (1:1000) |
| **ATG5** | Abclonal A19677 | WB (1:1000) |
| **LAMP1** | Invitrogen MA1-164 | WB (1:500) |
| **LAMP2** | Santa Cruz sc-71492 | WB (1:200) |
| **Ac-K-100** | CST 6952S | WB (1:1000) |
| **MFN1** | Abcam ab221661 | WB (1:1000) |
| **MFN2** | proteintech 12186-1-AP | WB (1:1000) |
| **OPA1** | proteintech 12957-1-AP | WB (1:1000) |
| **DRP1** | CST 8570S | WB (1:1000) |
| **Donkey anti-Mouse IgG (H+L), Alexa Fluor™ 488** | Invitrogen A-21202 | IF (1:500) |
| **Donkey anti-Rabbit IgG (H+L), Alexa Fluor™ 647** | Invitrogen A-31573 | IF (1:500) |

**Table S2. Western blot antibodies for related experiments**

| **Target name** | **Forward sequence** | **Reverse sequence** |
| --- | --- | --- |
| **Mouse Hint2-si** | AGAUGGGUAUCGACUUGUGGUUAAUTT | AUUAACCACAAGUCGAUACCCAUCUTT |
| **Human Hint2-si-1** | AGAAGCCCAUUCCUCGGAUUA | UAAUCCGAGGAAUGGGCUUCU |
| **Human Hint2-si-2** | GGGAGAUGGAUACCGACUUGU | ACAAGUCGGUAUCCAUCUCCC |
| **Human Hint2-si-3** | CUGGGUGCACAAUCUGUGUAU | AUACACAGAUUGUGCACCCAG |

**Table S3. Primer sequence of si*Hint2* for NMVM and 293T**

| Primer | Primer Seqence(5’-3’) F | Primer Seqence(5’-3’) R |
| --- | --- | --- |
| primer 1: promoter(-1974, -1765) | TGTGCATCTTACCAAGAGAAGGAC | TCTCTAGTCCCATGACTTCAGC |
| primer 2: promoter(-1370, -1120) | TGCTACTTTGCCTCACCTTTGG | TCTCTGGAAGTAGGACTAAGCC |
| primer 3: promoter(-1140, -909) | CCTACTTCCAGAGAGCCAATGT | TTTCTGTATAGGCAATGGCATGA |
| primer 4: promoter(-988, -772) | ACTGGTATTTAAGGGCTGGAAGG | CCCTCTGCAAACGAGGATGTTA |
| primer 5: promoter(-792, -543) | AACATCCTCGTTTGCAGAGGG | ACAAGTTCTAATGTCGGTGGTCT |
| primer 6: promoter(-570, -368) | TCTCAAGACCACCGACATTAGAAC | GGAGCGTCTCAGTTCAAGCCA |
| primer 7: promoter(-386, -118) | TGAACTGAGACGCTCCGC | AAACCGCAATCCTCTAGCCTG |

**Table S4. Primers of *TFAM* promoter for ChIP PCR assay**
